# A scoping review of the application scope of digital technologies in lower limb rehabilitation and balance training for children with cerebral palsy

**DOI:** 10.3389/fped.2026.1786311

**Published:** 2026-03-18

**Authors:** Yang Wang, Yinhu Tan, Xiaoli Zheng, Mengyao Wang, Hang Li, Xiuling Zhou

**Affiliations:** 1 School of Nursing, Changchun University of Chinese Medicine, Changchun, Jilin, China; 2 Surgical Treatment Area, The Third Affiliated Hospital of Changchun University of Chinese Medicine, Changchun, Jilin, China

**Keywords:** cerebral palsy, children, digital technologies, internet, robotic, VR

## Abstract

**Objective:**

This study aims to systematically review the application scope of digital technologies in lower limb rehabilitation and balance training for children with cerebral palsy (CP), identify the intervention effects and barriers associated with various types of technologies, and provide evidence-based guidance for clinical practice and home-based rehabilitation.

**Methods:**

Following the Joanna Briggs Institute (JBI) scoping review framework, a comprehensive literature search was conducted in PubMed, Embase, Web of Science, and the Cochrane Library for studies published from January 1, 2008, to January 1, 2025. The included studies were published between 2008 and 2024. Eligible studies included children with CP aged ≤18 years, involving the application of digital technologies in any form to lower limb or balance-related assessment, intervention, validation, or prediction Two reviewers independently screened the literature and extracted valid data. Study quality was assessed using the Mixed Methods Appraisal Tool (MMAT).

**Results:**

A total of 85 studies were included, encompassing five major categories of digital technologies: robotics/exoskeletons, virtual reality, sensors with real-time feedback, internet-based telerehabilitation, and game-based technologies. Robotics and exoskeletons significantly improved gait and balance in children through repetitive and precise training. Virtual reality and game-based interventions enhanced postural control, motor engagement, and motivation. Sensor and feedback technologies emphasize real-time monitoring and personalized correction. Internet platforms showed potential value in home-based rehabilitation and functional training. However, high costs, technical complexity, and limited cultural adaptability remain major barriers to widespread adoption and implementation.

**Conclusion:**

Digital technologies have been evaluated for lower-limb and balance rehabilitation in children with CP, and several studies reported improvements in gait- and balance-related outcomes and training engagement. Future research should strengthen methodological rigor, cost-effectiveness evaluation, and long-term follow-up to better inform implementation and equity considerations.

**Systematic Review Registration:**

https://doi.org/10.17605/OSF.IO/TMK93, identifier TMK93.

## 1 Introduction

Cerebral palsy (CP) is a neurodevelopmental disorder caused by non-progressive brain injury occurring in the developing fetal or infant brain. It is characterized by impairments in motor, sensory, cognitive, and perceptual functions. CP frequently results in secondary musculoskeletal complications (1), reduced walking ability and impaired balance (2), and substantial limitations in activities of daily living and overall quality of life (3–5) The global prevalence of CP varies across socioeconomic contexts, affecting approximately 1.4–2.5 per 1,000 live births in high-income countries, with even higher rates reported in low- and middle-income countries (6). Lower limb mobility and postural balance are key determinants of functional independence in children with CP. Reduced gait speed and impaired balance not only restrict community participation and quality of life but also increase the risk of falls (7–9) Optimizing lower limb function and balance is therefore a central goal of pediatric neurorehabilitation.

Traditional rehabilitation approaches—including physical therapy, orthotic interventions, and task-oriented training—remain the mainstay of treatment. Although beneficial, these methods often require prolonged intervention periods, rely heavily on therapist expertise, and may be associated with variable adherence and inconsistent outcomes. These limitations highlight the need for innovative adjunctive strategies that can enhance rehabilitation efficiency, engagement, and accessibility.

In recent years, digital technologies—including computer-based systems, information and communication technologies (ICTs), artificial intelligence, robotics, and data-driven platforms—have emerged as promising tools in pediatric neurorehabilitation (10). Virtual reality (VR), for example, integrates therapeutic objectives into immersive and interactive environments to enhance motivation and adherence to high-intensity training (11). Robotic and exoskeleton devices provide task-specific gait assistance and have demonstrated improvements in gait speed and distance compared with conventional treadmill-based therapy (12). Robotic and exoskeleton devices provide task-specific gait assistance and have demonstrated improvements in gait speed and distance compared with conventional treadmill-based therapy (13).

Although a growing body of empirical research has investigated various digital technologies in lower limb and balance rehabilitation, existing reviews have largely focused on specific technologies or predominantly synthesized upper-limb outcomes (14–17). To date, the overall application scope of digital technologies targeting lower limb function and balance in children with CP has not been comprehensively synthesized. Therefore, this scoping review systematically summarizes evidence from studies published between 2008 and 2024 (search conducted through January 1, 2025) to examine the range of digital technologies applied in lower limb and balance-related contexts in children with CP. In addition, it explores the multilayered barriers to their integration into clinical and home-based rehabilitation. By providing a structured synthesis of available evidence, this review aims to inform clinical decision-making and guide future research priorities, in alignment with the WHO Global Digital Health Strategy and its emphasis on universal health coverage (18).

## 2 Methods

This review was conducted following the methodological framework outlined in the scoping review guidelines issued by the Joanna Briggs Institute (JBI), Australia (19). The study protocol was prospectively registered on the Open Science Framework (OSF) at http://osf.io, with the registration number: https://doi.org/10.17605/OSF.IO/TMK93. The PRISMA-ScR (Preferred Reporting Items for Systematic reviews and Meta-Analyses extension for Scoping Reviews) checklist was used for reporting.

### 2.1 Identifying the research question

Based on a comprehensive literature review and group discussion, the primary research questions of this study were identified as follows: (1) What types of digital technologies are used in lower limb and balance-related contexts for children with CP, and what are their application outcomes? (2) What barriers and facilitators influence the implementation of digital technologies in this context?

### 2.2 Literature search

Systematic search was conducted in the PubMed, Embase, Web of Science, and Cochrane Library databases. The search strategy combined Medical Subject Headings (MeSH), free-text terms, and Boolean logic operators. Citation tracking was also performed. The search covered literature published from January 1, 2008, to January 1, 2025. No language restrictions were applied during the search. Search terms were organized into three concept blocks: (1) cerebral palsy, (2) children, and (3) digital technology. In PubMed, each block included both MeSH terms and Title/Abstract keywords. Synonyms within each block were combined with OR, and the three blocks were combined with AND. Specifically, the cerebral palsy block combined Cerebral Palsy[MeSH] with free-text terms (e.g., CP, spastic diplegia, atonic/hypotonic/dyskinetic cerebral palsy, dystonic-rigid and mixed cerebral palsies) using OR; the children block combined Child[MeSH] and Disabled Children[MeSH] with free-text terms (e.g., handicapped children, children with disabilities, children) using OR; and the digital-technology block combined MeSH terms (Digital Technology, Internet, Virtual Reality, Exergaming, Computers, Wearable Electronic Devices, Robotics, Artificial Intelligence) with free-text terms (e.g., “information and communication technology” [Information AND Communication Technology], ICT, VR, AI, web, cyberspace, instructional/virtual reality exercise, active-video gaming, wearable devices/technology, socially assistive robots, telemedicine, telehealth, mHealth, intelligent robot, platform, videoconferencing) using OR. The final PubMed query was constructed as (cerebral palsy terms) AND (children terms) AND (digital technology terms). To maximize sensitivity, outcome-specific terms were not added at the search stage; relevance to lower-limb function and balance was applied during screening. The complete stepwise PubMed strategy is provided in Supplementary Appendix 1.

### 2.3 Inclusion and exclusion criteria

The inclusion and exclusion criteria for this study were developed based on the PICOS framework: population, intervention, comparison, outcomes, and study design. The inclusion criteria were as follows: (1) Population (P): children (≤18 years) with a diagnosis of cerebral palsy (CP) as reported/confirmed in the original studies (e.g., clinician diagnosis or medical records). No restrictions were applied to the diagnostic criteria or methods used to establish CP, given the descriptive scope of this review. Studies including mixed populations were eligible if CP-specific data could be extracted; (2) Intervention (I): studies involving the application of digital technologies in children with cerebral palsy, including but not limited to VR, augmented reality (AR), artificial intelligence (AI), robotics, big data, and computer-based applications. (3) Comparison (C): not specifically restricted; (4) Outcomes (O): studies reporting clinical outcomes, functional measures, or technology-related evaluation metrics associated with lower-limb function and/or balance/postural control in children with CP. (5) Study Design (S): randomized controlled trials, non-randomized quantitative studies (e.g., pre-post or quasi-experimental studies), quantitative descriptive studies (e.g., cross-sectional or validation studies), qualitative studies, and mixed-methods research. The exclusion criteria were: (1) study protocols, guidelines, opinion papers, or policy documents; (2) review articles; (3) duplicate publications without full-text.

### 2.4 Literature screening

Retrieved references were imported into EndNote X9 reference management software to remove duplicates. Two researchers (YW and YHT), both trained in evidence-based medicine, independently screened the studies based on the predefined inclusion and exclusion criteria. Initial screening was performed by reviewing titles and abstracts, followed by full-text screening. Any discrepancies were resolved through discussion or consultation with a third reviewer (XLZ).

### 2.5 Data extraction process

Two researchers (YW and YHT) independently extracted the data, cross-checked the results, and performed a consolidated analysis. Any disagreements were resolved by consultation with a third researcher (XLZ). Extracted data included the author(s) and year of publication, country, research design, study subjects, type of digital technology applied, intervention measures (where applicable), intervention frequency and duration, and the main research results. Detailed information on the extracted variables is provided in Supplementary Appendix 2.

### 2.6 Quality appraisal

Given the heterogeneity in study designs among the included literature, the Mixed Methods Appraisal Tool (MMAT) was selected for quality assessment (20). The MMAT encompasses five categories of study designs: qualitative research, quantitative randomized controlled trials, quantitative non-randomized studies, quantitative descriptive studies, and mixed methods research. It is widely used for assessing the methodological quality of mixed-design reviews. Based on the types of studies included, the checklist items from the “quantitative randomized controlled trials,” “quantitative non-randomized studies,” and “quantitative descriptive studies” sections of the MMAT were applied for evaluation. The MMAT includes two general screening questions and five criteria-specific questions. Studies that meet the screening criteria are eligible for further appraisal using the MMAT. Each criterion was rated as “★” for meeting the standard, “–” for not meeting the standard, and “/” for unclear. Overall methodological quality was summarized using the number of “★” and corresponding percentages. Quality assessment was independently conducted by two reviewers (MYW and HL) and cross-verified for consistency. Detailed quality assessment results for all included studies are presented in Supplementary Appendix 3. In line with the descriptive purpose of a scoping review, MMAT ratings were not used to exclude studies or to formally weight effect estimates. Instead, MMAT results were used to contextualize the narrative synthesis. Findings from studies meeting fewer MMAT criteria were interpreted with caution, and conclusions were drawn based on the overall consistency of evidence across studies. When summarizing patterns across technologies and outcomes, variations in methodological quality were acknowledged, and areas where evidence was limited or largely derived from studies with lower methodological rigor were explicitly noted to highlight evidence gaps and inform future research.

### 2.7 Data synthesis

Given the heterogeneity in study designs, intervention protocols, and outcome measures across the included studies, we conducted a descriptive summary and narrative synthesis rather than a meta-analysis. First, extracted study characteristics (e.g., publication year, country, study design, participant characteristics, and technology category) were summarized using descriptive statistics (counts and percentages) and presented in the text, tables, and figures. Second, consistent with the structure of the results, studies were grouped into five categories of digital technologies (robotics/exoskeletons, sensor-based technologies with real-time feedback, internet-based training, virtual reality, and game-based technologies). Within each category, we narratively synthesized reported applications, intervention components (where applicable), dose parameters (frequency, session duration, and program length), comparators, outcome measures, and key findings. Third, to address the second research question, information related to implementation barriers and facilitators reported in the included studies was collated and summarized thematically. Barriers and facilitators identified during data charting were organized into higher-order themes reflecting multi-level implementation challenges (e.g., cost and access, technical complexity and usability, safety considerations, setting and workforce requirements, adherence and engagement, cultural or contextual adaptability, and limitations of long-term evidence).

## 3 Results

**Figure 1:**
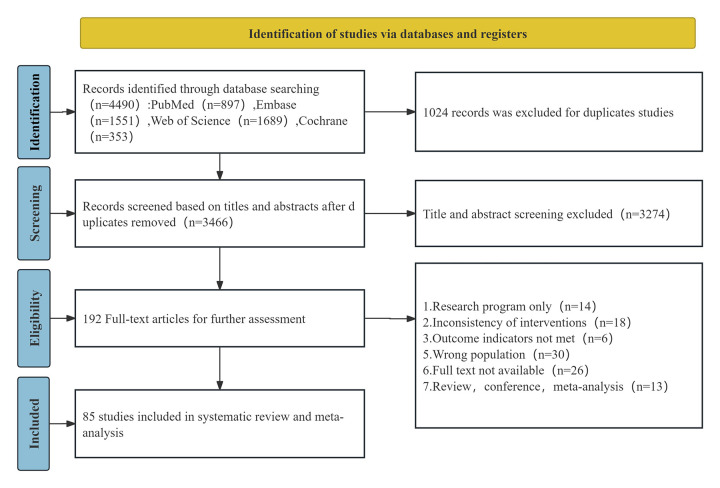
PRISMA flow diagram of study selection and inclusion process.

A total of 4,490 references were initially retrieved. After removing duplicates, 3,466 studies remained. Titles and abstracts were reviewed for initial screening, resulting in the exclusion of 3,274 studies. The remaining 192 articles were further screened by full-text review, and 107 studies were excluded for not meeting the inclusion and exclusion criteria. Ultimately, 85 studies were included (11, 13, 21–103). The literature selection process is depicted in Figure 1.

### 3.1 Quality of the literature

As recommended for scoping reviews, the MMAT results are reported to describe the methodological characteristics of the included evidence and to inform interpretation, rather than to determine study eligibility. All studies met the general screening criteria, and the MMAT was deemed applicable for quality assessment. Among the non-randomized quantitative studies, 10 studies (29.4%) met 60% of the MMAT criteria, 16 studies (47.1%) met 80% of the criteria, and 8 studies (23.5%) met all quality assessment criteria. Among the randomized controlled trials, 8 studies (30.8%) met 60% of the MMAT criteria, 15 studies (57.7%) met 80% of the criteria, and 3 studies (11.5%) met 100% of the quality assessment criteria. Among the quantitative descriptive studies, 10 studies (40.0%) met 60% of the MMAT criteria, 11 studies (44.0%) met 80% of the criteria, and 4 studies (16.0%) met 100% of the quality assessment criteria.

### 3.2 Publication year, country, and study type

**Figure 2:**
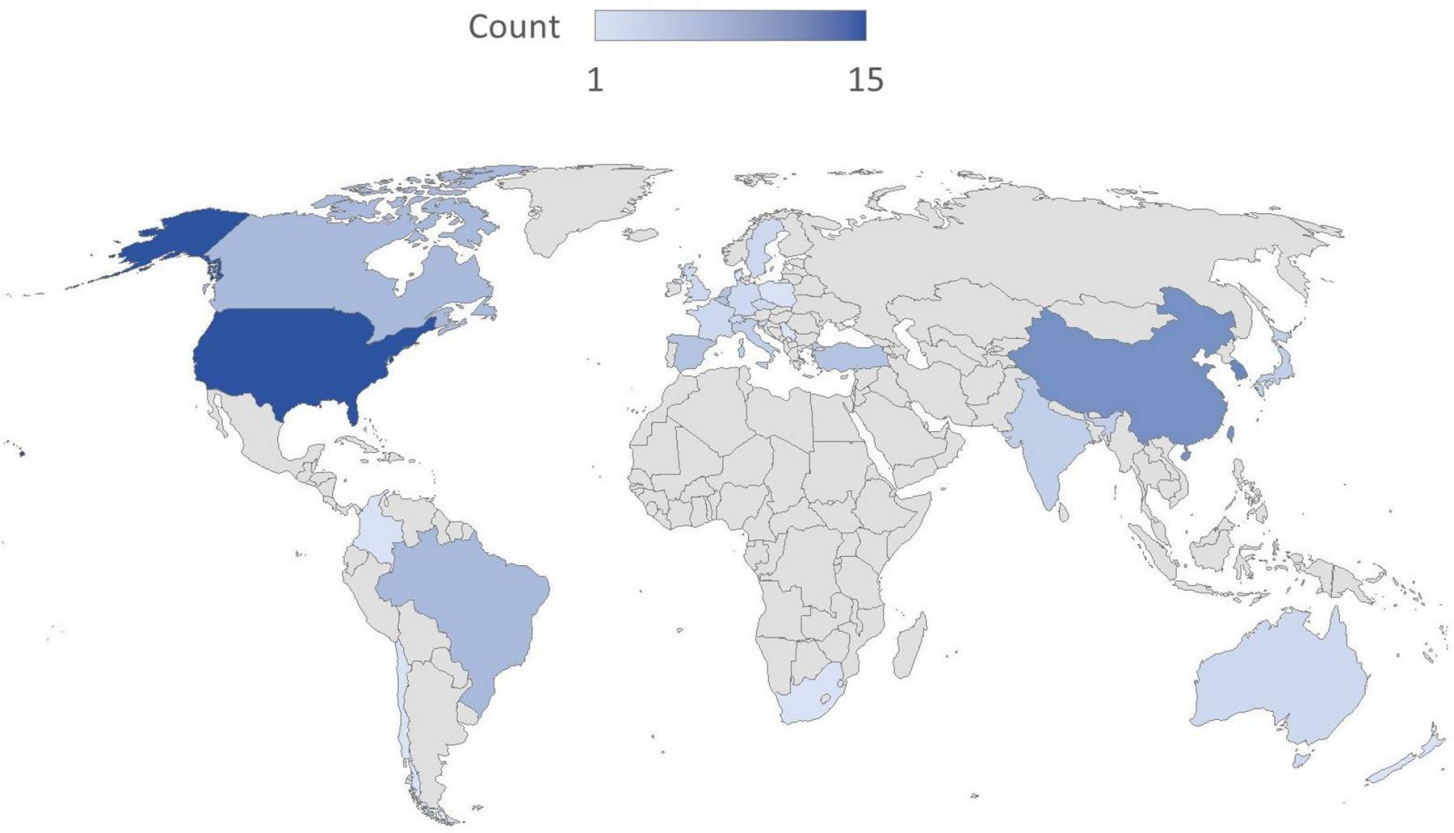
Global distribution of included studies by country.

**Figure 3:**
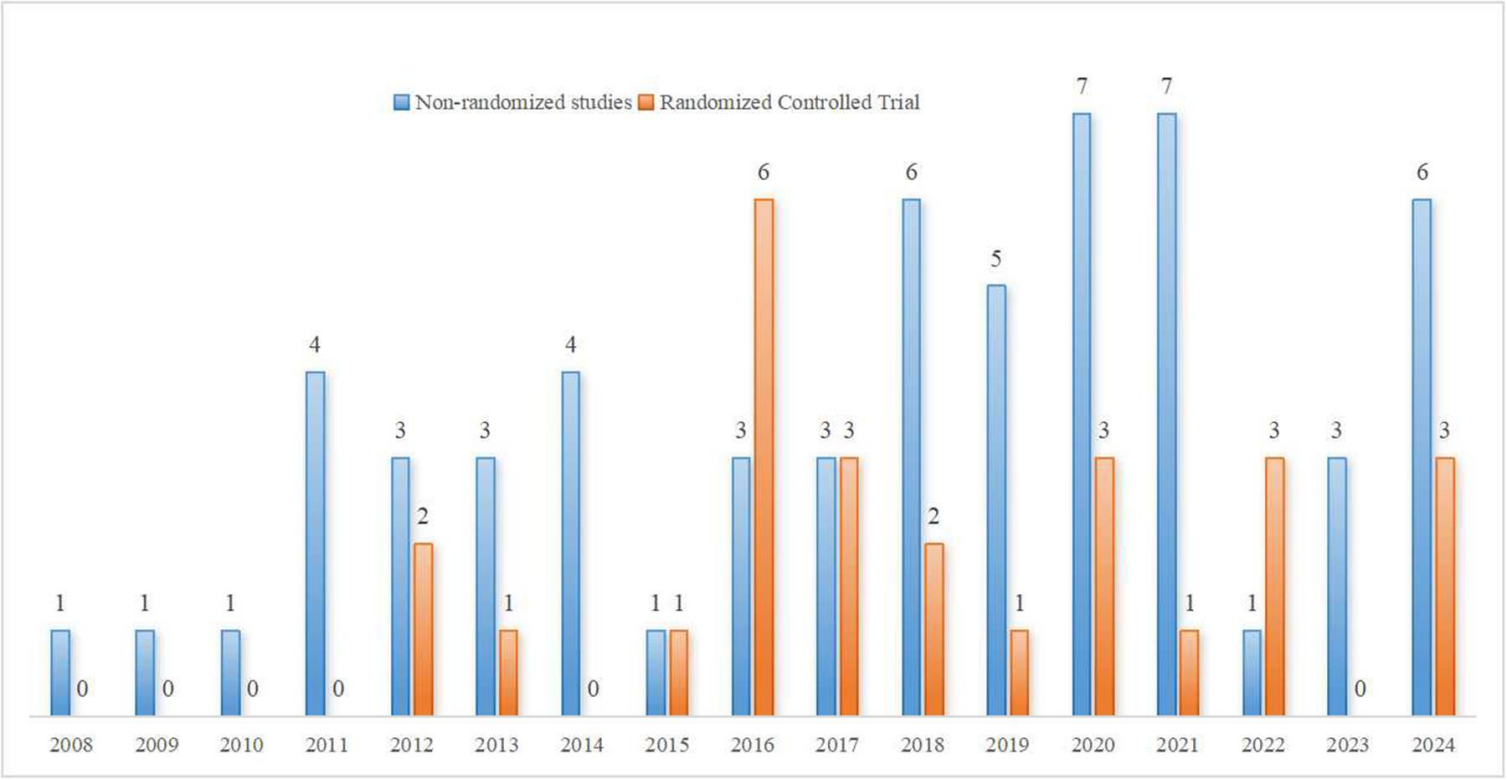
Annual publication trends by study design (non-randomized studies and randomized controlled trials), 2008–2024.

The publication period of the included studies spans from 2008 to 2024, although the search was conducted through January 1, 2025. Prior to 2016, fewer than five articles were published annually, but there was a significant increase in publications after 2016. Studies published between 2020 and 2024 accounted for 40% of the total (34/85). Among the countries of publication, 7 studies (22, 53, 62, 66, 77, 85, 101) involved international collaboration. For the remaining 78 single-country studies, high-income countries (HICs) accounted for the majority of research output, comprising 71.8% (56/78), while low- and middle-income countries (LMICs) accounted for 28.2% (22/78) (classified according to the World Bank income groups). The majority of studies came from the United States (15 studies) (21, 24, 30, 35, 38, 40, 42, 47, 49, 55, 56, 64, 80, 93, 95), South Korea(10 studies) (43, 44, 58, 72, 79, 82–84, 88, 97), Canada(5 studies) (23, 36, 61, 67, 90), Japan(3 studies) (73–75), Spain(4 studies) (34, 54, 96, 98), Australia(2 studies) (13, 26), Chile(1 studies) (103) and other European HICs (Switzerland, Netherlands, Belgium, etc., totaling 16 studies). LMIC studies were mainly contributed by China(7 studies) (31, 50, 57, 60, 71, 100, 102), India (3 studies) (11, 51, 78), Brazil (5 studies) (39, 41, 46, 63, 69), and Turkey (4 studies) (45, 68, 76, 87), as well as South Africa (33), Jamaica (28), and Colombia (70). Notably, China contributed 31.8% (7/22) of LMIC studies. As shown in Figure 2, the choropleth map summarizes the geographic distribution of the included studies by country. For studies involving international collaboration, each participating country was counted once (full counting). The study types include 59 non-randomized studies and 26 randomized controlled trials, with trends in study design shown in Figure 3.

### 3.3 Study population

The age range of the participants in the included studies was from 2 to 18 years old, with the 7–12 year age group being the primary focus of research. Sample sizes ranged from 1 to 356. In terms of functional classification, the Gross Motor Function Classification System (GMFCS) was widely used. Among the included studies, patients classified as GMFCS I-III predominated, with GMFCS II being the most frequently represented level. Furthermore, prospective studies targeting GMFCS IV-V patients have gradually increased since 2016. For example, Avaltroni et al. (94) used the Moonwalker exoskeleton to improve standing ability in children with severe motor impairments, indicating that the adaptability of the technology is expanding to a broader range of functional levels. At the same time, significant population adaptation characteristics were observed across different technology types. GMFCS I-II patients tended to prefer VR due to the high level of active participation required. Among GMFCS III-IV patients, the use of robotics and exoskeletons was more prevalent, as these technologies provided mechanical assistance to compensate for motor impairments.

### 3.4 Types of techniques and main research findings

**Figure 4:**
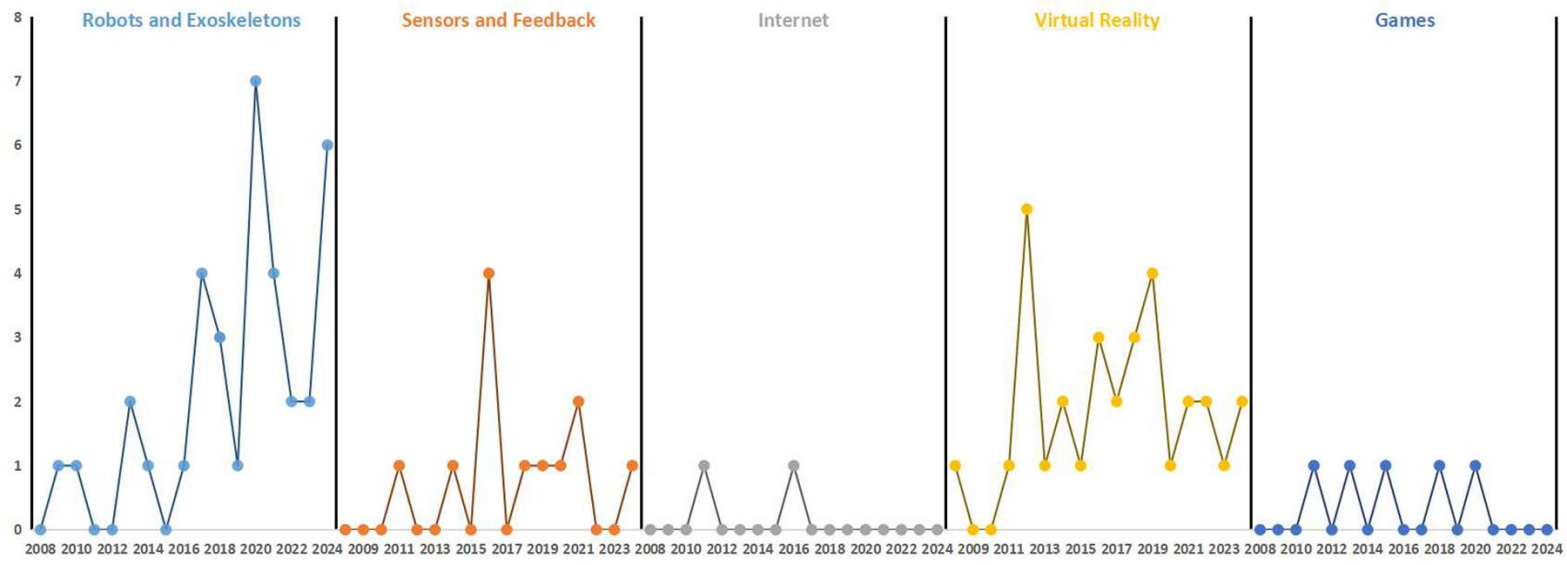
Temporal distribution of included studies by digital technology category (2008–2024).

The current studies involved various types of technologies, which were categorized into five types of digital technologies. Among these, 35 studies applied robotics and exoskeleton technology, 12 studies used sensor and feedback technologies, 2 studies involved internet-based rehabilitation training, 31 studies utilized VR technologies, and 5 studies applied game-based technologies. In terms of publication trends, robotics and exoskeleton technologies showed a steady growth, with a significant increase from 2016 to 2020. There was a slight decline from 2022 to 2024, though the application rate remained high. Sensor and feedback technologies were in a significant rise from 2013 to 2016, followed by fluctuations and a decline between 2019 and 2024. Internet and game-based technologies consistently had low and stable application frequencies. VR technology experienced rapid growth and peaked in 2012, followed by a fluctuating decline. The publication trends of different technology types are shown in Figure 4.

#### 3.4.1 Robotics and exoskeleton-related technologies

Robotics and exoskeleton technologies primarily utilize robotic-assisted gait training systems (e.g., Lokomat, HAL, Walkbot-K) and exoskeleton devices (e.g., CPWalker, Trexo Home). Intervention measures include passive joint stretching, active movement training, and robot-assisted gait training. The intervention frequency generally ranged from 3 to 5 sessions per week, with each session lasting 45–60 min. The duration of the interventions varied from 3 to 6 weeks. The main findings indicated that robotics and exoskeleton technologies significantly improved gait speed, range of motion, and motor control abilities in children with CP. For instance, Meyer-Heim et al. (22) found that DGO robotic-assisted treadmill training effectively improved the GMFM standing dimension and significantly enhanced 6-minute walk test (6 MinWT) performance. Wu et al. (24) demonstrated that portable robotic training significantly improved selective motor control and both passive and active range of motion. Additionally, several studies (68, 92) showed that the intervention effects were sustained even after three months. Notably, the study by Sucuoglu et al. (76) indicated that improvements in children with mild to moderate CP (GMFCS I-III) were significantly greater than those in severe cases, suggesting that the applicability of the technology should be considered in relation to functional classification.

#### 3.4.2 Sensor-based and feedback-related technologies

Most sensor and feedback devices (e.g., ActiGraph accelerometers, inertial sensors, gyroscope sensors, motion capture systems, and sensor-based gait analysis platforms) are applied in gait analysis. Several studies have used these devices to assess gait parameters in children with CP, revealing significant differences in gait speed, stride length, and gait symmetry compared to healthy controls, with high assessment accuracy (37, 69, 78). Additionally, some studies have utilized these devices for gait training, showing that feedback training significantly improved gait quality, particularly in terms of gait speed, stride length, and gait symmetry. For example, the study by Pu et al. (50) found that real-time feedback training significantly improved heel strike impact and the percentage of normal gait, outperforming traditional gait training methods. Behboodi et al. (64) used a gyroscope and sensor system for real-time gait analysis, triggering Functional Electrical Stimulation (FES) to enhance gait reliability and stability. Behboodi et al. (95) combined Brain-Computer Interface-Neurofeedback Training (BCI-NFT) with Electroencephalogram (EEG) and Neuromuscular Electrical Stimulation (NMES) to train ankle dorsiflexion. They found significant improvements in ankle dorsiflexion speed, as well as increases in gait speed, stride length, and cadence after training. EEG analysis showed enhanced brain activity, indicating improved neuroplasticity, highlighting the potential of BCI-NFT-based feedback technologies in the rehabilitation of children with CP.

#### 3.4.3 Internet-based technologies

Internet technology supports home-based personalized training for children with CP, with platforms providing remote guidance and data tracking, focusing on functional independence and self-management skills. Studies have shown that internet-based training significantly improves lower limb strength and endurance, and when combined with cognitive training, it can also enhance visuomotor integration abilities (25). However, the study by Mitchell et al. (13) indicated that internet-based training did not translate into enhanced activity performance, increased recreational participation, or reduced activity limitations. Although evidence on lower-limb and balance telerehabilitation in children with CP remains limited, internet-based approaches may help mitigate resource constraints in underserved regions. With ongoing advances in connectivity and remote monitoring, home-based rehabilitation may play a growing role, but its feasibility, accessibility, and clinical effectiveness require further evaluation in pediatric CP.

#### 3.4.4 VR-related technologies

VR technology is widely used in lower limb rehabilitation training for children with CP. The training frequency for different VR systems typically ranges from 2 to 5 sessions per week, with session durations generally between 20 and 45 min, and the intervention period typically lasts from 3 to 12 weeks. Several studies have used devices such as Wii Fit, Xbox Kinect, and virtual cycling training systems, combined with various VR games and exercises, for rehabilitation training. For example, Deutsch et al. (21) used the Wii Sports game for rehabilitation training and found that patients showed improvements in visual-perceptual processing, postural control, and functional activity abilities. Brien et al. (23) conducted balance training using a VR system and found significant improvements in balance function and gait ability in adolescents, with the effects lasting for up to one month. Jelsma et al. (33) used the Nintendo Wii Fit for balance and motor training, which significantly improved balance scores, although other evaluation metrics did not show significant differences. Several studies have combined VR with other therapeutic approaches. For example, Collange Grecco et al. (41) combined VR gait training with anodal tDCS, which significantly improved gait speed, gait cadence, and gross motor function. This effect was maintained one month after the intervention. Additionally, Luna-Oliva et al. (34) combined Xbox 360 Kinect with physical therapy, resulting in significant improvements in GMFM, AMPS, PRT, and 10 MW in all participants. These improvements were sustained for 8 weeks. Overall, several studies reported improvements in gait- and balance-related outcomes following VR-based interventions; however, findings were mixed across outcomes and study designs. VR-based training may support engagement and motivation, but long-term effectiveness and generalizability remain uncertain. Some studies have further highlighted the synergistic effects of combining VR with other training methods, which further improved training outcomes.

#### 3.4.5 Game-related technologies

Unlike VR games, game-based technologies use non-immersive video or computer games (e.g., Balance Master, PlayStation EyeToy) to promote motor learning and energy expenditure through interactive tasks. Sandlund et al. (27) used interactive game training with PlayStation 2 and EyeToy, and found that after the intervention, children's motor performance and activity levels significantly increased, particularly in terms of improved motivation and active participation from both parents and children. However, no significant progress was observed in the 1-minute walk test and the BOTMP 5:6 test. Radtka et al. (35) showed that video game-based physical therapy enhanced the appeal of balance training. The system demonstrated good safety and ease of use, making it suitable for home-based use by children. Bingham et al. (40) used the Balance Master postural tracing game to assess balance control in children with CP. The results showed a significant correlation between balance game performance and GMFM scores, particularly in the improvement of directional control and balance ability. Game-based technologies were reported to improve selected motor outcomes and may increase participation and motivation; their feasibility for home use appears promising but requires further evaluation. Notably, a study (27) indicated that, in most cases, children are more likely to engage in games that involve playing with others and include competitive elements, suggesting future research directions for game-based technologies.

### 3.5 Implementation barriers and facilitators

Across the included studies, barriers and facilitators affecting the implementation of digital technologies were reported at multiple levels (technology, user, setting, and system). Commonly reported barriers included: (1) limited economic accessibility and high equipment costs, particularly for high-end robotic systems; (2) technical complexity/usability and safety concerns, especially when children required supervision and individualized decision-making during training; (3) setting and workforce constraints, as most studies were conducted in hospitals/laboratories/treatment centers, with relatively few implemented at home or in schools; (4) limited cultural/contextual adaptability of some digital content (e.g., VR scenarios); and (5) insufficient long-term evidence, with limited follow-up durations and less frequent reporting of broader outcomes such as quality of life and psychosocial impacts. Facilitators described in the literature included features that improved engagement and adherence (e.g., interactive and game-like designs), real-time monitoring/feedback that supported individualized correction, and remote guidance and data tracking that enabled home-based training for some families.

## 4 Discussion

The application of digital technologies in lower limb rehabilitation and balance training for children with CP has gradually become a research hotspot. Digital technologies may enhance rehabilitation engagement and participation, and could support more intensive and feedback-driven practice in some settings. Current applications encompass five major types of technology: VR, robotics and exoskeletons, sensors and feedback, internet and game-based technologies. In terms of study types, early research (2008–2015) largely focused on exploratory non-randomized designs. In recent years (2016–2024), the proportion of randomized controlled trials (RCTs) has increased to 36.1% (22/61), reflecting a shift toward more evidence-based research. Although most studies used group samples, 7 studies (21, 39, 47, 70, 74, 90, 95) (8.2%) employed single-case designs, which primarily focused on exploring the individualized intervention effects in specific subtypes or patients with severe functional impairments. It is important to note that CP encompasses multiple types (e.g., ataxia, choreoathetosis). However, in the studies included in this research, most of the populations were simply classified as children with CP without specific subtype differentiation. Different types of CP exhibit different clinical manifestations and prognoses. In clinical practice, treatment plans for most children with CP do not differ significantly. Cho et al. (43) also pointed out that future research may focus on a more in-depth exploration of the specific application effects for different types of CP. Therefore, it is crucial to identify the clinical characteristics of children with different subtypes of CP and adopt targeted digital treatment plans.

Despite the rapid development of technologies, their clinical application still faces multiple challenges. Limited economic accessibility is the primary barrier. Research conducted in low- and middle-income countries (LMICs; classified according to the World Bank income groups) has increased since 2016; however, substantial geographic disparities persist, with high-income countries (HICs) accounting for the majority of single-country studies, and evidence from many resource-limited and rural settings remaining scarce. The cost of high-end equipment (e.g., Lokomat robots) can reach several hundred thousand dollars, making it unaffordable for low-income families and resource-limited institutions. Technological complexity and issues related to user adaptability represent important implementation challenges. Deutsch et al. (21) noted that unsupervised training may be inadvisable and potentially unsafe for some children with CP, particularly those with cognitive impairments. In routine rehabilitation practice, important clinical decisions typically require therapist supervision and individualized adjustments. However, in multidisciplinary clinical settings, children with CP may attend rehabilitation services less frequently (51), which may limit opportunities for continuous professional monitoring.

In addition, most studies included in this review were conducted in hospitals, laboratories, or specialized treatment centers, with only a few implemented in home or school environments (13, 38, 46, 61). This discrepancy between research settings and real-world practice highlights the need to develop safe and reliable home-based or portable rehabilitation solutions. Hybrid models that combine digital training (e.g., exergaming/VR or sensor-based feedback systems) with telecoaching or remote supervision may offer a potential strategy to support individualized progression, safety monitoring, and sustained engagement. Evidence from broader neurological rehabilitation literature suggests that telecoaching-supported programs may be feasible and could improve adherence and functional outcomes, although findings remain heterogeneous (104, 105). Such models may help mitigate several implementation barriers identified in this review, including limited access to specialist therapists, travel burden, insufficient real-time feedback in home settings, and challenges in maintaining long-term adherence. Nevertheless, rigorous studies in pediatric CP populations remain necessary, incorporating standardized outcome measures, longer follow-up durations, and implementation-focused evaluations addressing feasibility, acceptability, caregiver burden, and cost-effectiveness.

Beyond structural and service-delivery challenges, cultural adaptability also influences the broader applicability of digital technologies. Many existing systems, particularly VR-based platforms, are developed within Western cultural contexts (e.g., skiing or golf scenarios), which may limit relevance and engagement in non-Western regions. Furthermore, long-term efficacy evidence remains limited. Only two studies tracked outcomes for more than five months (25, 84), and broader outcome domains—such as quality of life (CP-QOL), psychosocial well-being, self-efficacy, and caregiver burden—were infrequently assessed, constraining conclusions regarding sustainability and real-world impact.

To address these challenges, future efforts may focus on three interrelated areas: technological innovation, policy collaboration, and cultural inclusivity. From a technological perspective, multimodal integration (e.g., VR combined with robotics or sensors integrated with AI-based feedback) and the development of scalable, lower-cost solutions may enhance accessibility. For example, open-source sensors and modular exoskeleton designs may reduce economic barriers, while home-oriented devices (e.g., Trexo Home systems) may expand opportunities for community-based rehabilitation. At the policy level, incorporating digital rehabilitation into health insurance coverage and strengthening international collaboration under global digital health initiatives (e.g., the WHO Digital Health Strategy) may help reduce regional disparities. In terms of cultural adaptability, collaboration with local communities to design contextually relevant training scenarios and offline-compatible modules may improve usability in regions with unstable internet infrastructure. At the same time, longer-term follow-up studies and real-world implementation research are needed to clarify sustainability, scalability, and equity implications, particularly for children and families in resource-limited settings.

## 5 Limitations

Although this study followed the methodological framework of a Scoping Review and systematically mapped the current state of digital technology applications in lower limb rehabilitation and balance training for children with CP, the following limitations should be noted: First, as the goal of a scoping review is to provide an overview of the research field and identify evidence gaps, rather than rigorously quantifying intervention effects or inferring causal relationships, the included studies exhibit heterogeneity in research design, rehabilitation protocols, and outcome measures, making it difficult to conduct strict comparisons and synthesis of specific intervention effectiveness. Second, some of the included studies lacked detailed reporting on long-term follow-up and blinding procedures, making it difficult to clarify the sustainability of the reported effects and the underlying mechanisms. In addition, much of the current evidence is derived from small-scale, non-randomized, or descriptive studies, which limits the strength and generalizability of the conclusions.

Despite these limitations, this scoping review has several strengths. We conducted a comprehensive multi-database search and followed PRISMA-ScR guidance. We systematically synthesized the range of digital technologies, outcome domains, and reported implementation barriers in lower-limb and balance-related contexts for children with cerebral palsy, providing an up-to-date overview of the available evidence to inform future trials and clinical translation.

## 6 Conclusion

Digital technologies have been increasingly explored for lower-limb rehabilitation and balance training in children with CP, and the existing literature suggests potential benefits for selected outcomes. However, barriers related to cost, technical complexity, cultural adaptability, and limited long-term evidence may constrain broader implementation. Future research should prioritize rigorous study designs, standardized outcomes, longer follow-up, and implementation-focused evaluations to determine feasibility and equity of access across diverse settings.

## Data Availability

The original contributions presented in the study are included in the article/Supplementary Material, further inquiries can be directed to the corresponding author.

## Supplementary material

The Supplementary Materialfor this article can be found online at: https://www.frontiersin.org/articles/10.3389/fped.2026.1786311/full#supplementary-material
