# Supplementary material for: A scoping review of the application scope of digital technologies in lower limb rehabilitation and balance training for children with cerebral palsy
Source: Front Pediatr. 2026 Mar 18;14:1786311. doi: 10.3389/fped.2026.1786311 (PMC13039068; doi:10.3389/fped.2026.1786311)
Supplement: Supplementary file 1 [file Supplementaryfile1.docx]

| **Research** | **General Screening Questions** | | **Targeting the Problem** | | | | | **Overall Score** | **Total Percentage** |
| --- | --- | --- | --- | --- | --- | --- | --- | --- | --- |
|  | S1 | S2 | Q1 | Q2 | Q3 | Q4 | Q5 |  |  |
| Sharan et al. (2012) | ★ | ★ | ★ | ★ | ★ | — | ★ | ★★★★ | 80% |
| Chen et al. (2012) | ★ | ★ | ★ | ★ | ★ | — | ★ | ★★★★ | 80% |
| Druz Bicki et al. (2013) | ★ | ★ | — | ★ | ★ | — | ★ | ★★★ | 60% |
| Collange Grecco et al. (2015) | ★ | ★ | ★ | ★ | ★ | ★ | ★ | ★★★★★ | 100% |
| Tarakci et al. (2016) | ★ | ★ | ★ | ★ | ★ | — | ★ | ★★★★ | 80% |
| Saxena et al. (2016) | ★ | ★ | ★ | ★ | ★ | ★ | ★ | ★★★★★ | 100% |
| Mitchell et al. (2016) | ★ | ★ | ★ | ★ | ★ | — | ★ | ★★★★ | 80% |
| Chen et al. (2016) | ★ | ★ | — | ★ | ★ | — | ★ | ★★★ | 60% |
| Lazzari et al. (2016) | ★ | ★ | ★ | ★ | ★ | ★ | ★ | ★★★★★ | 100% |
| Cho et al. (2016) | ★ | ★ | ★ | ★ | ★ | — | ★ | ★★★★ | 80% |
| Wallard et al. (2017) | ★ | ★ | ★ | — | ★ | — | ★ | ★★★ | 60% |
| Gatica-Rojas et al. (2017) | ★ | ★ | ★ | ★ | — | ★ | — | ★★★ | 60% |
| Wu et al. (2017) | ★ | ★ | ★ | ★ | ★ | — | ★ | ★★★★ | 80% |
| Wallard et al. (2018) | ★ | ★ | ★ | ★ | ★ | — | ★ | ★★★★ | 80% |
| Hsieh et al. (2018) | ★ | ★ | ★ | ★ | ★ | — | ★ | ★★★★ | 80% |
| Arnoni et al. (2019) | ★ | ★ | ★ | ★ | ★ | — | ★ | ★★★★ | 80% |
| Hsieh et al. (2020) | ★ | ★ | — | ★ | ★ | — | ★ | ★★★ | 60% |
| Kawasaki et al. (2020) | ★ | ★ | — | ★ | ★ | — | ★ | ★★★ | 60% |
| Jin et al. (2020) | ★ | ★ | ★ | ★ | ★ | — | ★ | ★★★★ | 80% |
| Farr et al. (2021) | ★ | ★ | ★ | ★ | ★ | — | ★ | ★★★★ | 80% |
| Gercek et al. (2022) | ★ | ★ | — | ★ | ★ | — | ★ | ★★★ | 60% |
| Jung et al. (2022) | ★ | ★ | — | ★ | ★ | — | ★ | ★★★ | 60% |
| Moll et al. (2022) | ★ | ★ | ★ | ★ | ★ | — | ★ | ★★★★ | 80% |
| Choi et al. (2024) | ★ | ★ | ★ | ★ | ★ | ★ | ★ | ★★★★ | 80% |
| Zhang et al. (2024) | ★ | ★ | ★ | ★ | ★ | — | ★ | ★★★★ | 80% |
| Hui et al. (2024) | ★ | ★ | ★ | ★ | ★ | — | ★ | ★★★★ | 80% |

**Randomized Controlled Trial (RCT)**

**Note：S1: Is there a clearly defined research question? S2: Is the collected data sufficient to answer the research question?**

**Q1 = Was randomization appropriately performed?
Q2 = Was allocation concealment adequately implemented?
Q3 = Were the groups comparable at baseline?
Q4 = Were outcome assessors blinded to the intervention provided?
Q5 = Did the participants adhere to the assigned intervention?**

**Non-randomized Quantitative Studies**

| **Research** | **General Screening Questions** | | **Targeting the Problem** | | | | | **Overall Score** | **Total Percentage** |
| --- | --- | --- | --- | --- | --- | --- | --- | --- | --- |
|  | S1 | S2 | Q1 | Q2 | Q3 | Q4 | Q5 |  |  |
| Meyer-Heim et al. (2009) | ★ | ★ | — | ★ | ★ | — | ★ | ★★★ | 60% |
| Wu et al. (2010) | ★ | ★ | — | ★ | ★ | — | ★ | ★★★ | 60% |
| Sandlund et al. (2011) | ★ | ★ | — | ★ | ★ | — | ★ | ★★★ | 60% |
| Bilde et al. (2011) | ★ | ★ | — | ★ | ★ | — | ★ | ★★★ | 60% |
| Brien et al. (2011) | ★ | ★ | — | ★ | ★ | ★ | ★ | ★★★★ | 80% |
| Jelsma et al. (2012) | ★ | ★ | — | ★ | ★ | ★ | ★ | ★★★★ | 80% |
| Gordon et al. (2012) | ★ | ★ | — | ★ | ★ | — | ★ | ★★★ | 60% |
| Ramstrand et al. (2012) | ★ | ★ | — | ★ | ★ | — | ★ | ★★★ | 60% |
| Luna-Oliva et al. (2013) | ★ | ★ | — | ★ | ★ | — | ★ | ★★★ | 60% |
| Chen et al. (2014) | ★ | ★ | — | ★ | ★ | — | ★ | ★★★ | 60% |
| Bingham et al. (2015) | ★ | ★ | ★ | ★ | ★ | — | ★ | ★★★★ | 80% |
| van Gelder et al. (2016) | ★ | ★ | ★ | ★ | ★ | — | ★ | ★★★★ | 80% |
| Pu et al. (2016) | ★ | ★ | ★ | ★ | ★ | — | ★ | ★★★★ | 80% |
| Chen et al. (2017) | ★ | ★ | ★ | ★ | ★ | — | ★ | ★★★★ | 80% |
| Meyns et al. (2017) | ★ | ★ | ★ | ★ | ★ | — | ★ | ★★★★ | 80% |
| Choi et al. (2018) | ★ | ★ | ★ | ★ | ★ | — | ★ | ★★★★ | 80% |
| Bulea et al. (2018) | ★ | ★ | ★ | ★ | ★ | ★ | ★ | ★★★★★ | 100% |
| Levac (2018) | ★ | ★ | ★ | ★ | ★ | — | ★ | ★★★★ | 80% |
| Booth et al. (2019) | ★ | ★ | ★ | ★ | ★ | ★ | ★ | ★★★★★ | 100% |
| Behboodi et al. (2019) | ★ | ★ | — | ★ | ★ | ★ | ★ | ★★★★ | 80% |
| Ma et al. (2019) | ★ | ★ | ★ | ★ | ★ | ★ | ★ | ★★★★★ | 100% |
| Yazıcı et al. (2019) | ★ | ★ | ★ | ★ | ★ | — | ★ | ★★★★ | 80% |
| Mills et al. (2019) | ★ | ★ | — | ★ | ★ | — | ★ | ★★★ | 60% |
| Sucuoglu(2020) | ★ | ★ | ★ | ★ | ★ | ★ | ★ | ★★★★★ | 100% |
| Barreira et al. (2020) | ★ | ★ | ★ | ★ | ★ | ★ | ★ | ★★★★★ | 100% |
| Jung et al. (2020) | ★ | ★ | ★ | ★ | ★ | ★ | ★ | ★★★★★ | 100% |
| Kim et al. (2021) | ★ | ★ | ★ | ★ | ★ | ★ | ★ | ★★★★★ | 100% |
| De Luca et al. (2022) | ★ | ★ | ★ | ★ | ★ | — | ★ | ★★★★ | 80% |
| Grodon et al. (2023) | ★ | ★ | ★ | ★ | ★ | — | ★ | ★★★★ | 80% |
| Lee et al. (2023) | ★ | ★ | ★ | ★ | ★ | — | ★ | ★★★★ | 80% |
| Avaltroni et al. (2024) | ★ | ★ | ★ | ★ | ★ | — | ★ | ★★★★ | 80% |
| Behboodi et al. (2024) | ★ | ★ | — | ★ | ★ | — | ★ | ★★★ | 60% |
| Castro et al. (2024) | ★ | ★ | ★ | ★ | ★ | ★ | — | ★★★★ | 80% |
| De Mulder et al. (2024) | ★ | ★ | ★ | ★ | ★ | ★ | ★ | ★★★★★ | 100% |

**Note：S1: Is there a clearly defined research question? S2: Is the collected data sufficient to answer the research question?**

**Q1 = Are the participants representative of the target population?
Q2 = Are measurements appropriate regarding both the outcome and the intervention (or exposure)?
Q3 = Are there complete outcome data?
Q4 = Are the confounders accounted for in the design and analysis?
Q5 = During the study period, is the intervention administered as intended?**

**Quantitative Descriptive Studies**

| **Research** | **General Screening Questions** | | **Targeting the Problem** | | | | | **Overall Score** | **Total Percentage** |
| --- | --- | --- | --- | --- | --- | --- | --- | --- | --- |
|  | S1 | S2 | Q1 | Q2 | Q3 | Q4 | Q5 |  |  |
| Deutsch et al. (2008) | ★ | ★ | ★ | — | ★ | ★ | ★ | ★★★★ | 80% |
| Clanchy et al. (2011) | ★ | ★ | ★ | ★ | ★ | ★ | ★ | ★★★★★ | 100% |
| Burdea et al. (2013) | ★ | ★ | — | — | ★ | ★ | ★ | ★★★ | 60% |
| Radtka et al. (2013) | ★ | ★ | — | — | ★ | ★ | ★ | ★★★ | 60% |
| Brégou Bourgeois et al. (2014) | ★ | ★ | ★ | — | ★ | ★ | ★ | ★★★★ | 80% |
| Pavão et al. (2014) | ★ | ★ | — | — | ★ | ★ | ★ | ★★★ | 60% |
| Ballaz et al. (2014) | ★ | ★ | ★ | — | ★ | ★ | ★ | ★★★★ | 80% |
| Lee et al. (2016) | ★ | ★ | ★ | ★ | — | — | ★ | ★★★ | 60% |
| Lerner et al. (2017) | ★ | ★ | ★ | ★ | — | ★ | ★ | ★★★★ | 80% |
| Gagliardi et al. (2018) | ★ | ★ | ★ | ★ | ★ | ★ | ★ | ★★★★★ | 100% |
| Chiu et al. (2018) | ★ | ★ | ★ | ★ | ★ | ★ | ★ | ★★★★★ | 100% |
| Bayón et al. (2018) | ★ | ★ | ★ | ★ | — | ★ | ★ | ★★★★ | 80% |
| Mataki et al. (2020) | ★ | ★ | ★ | ★ | — | ★ | ★ | ★★★★ | 80% |
| Kuroda et al. (2020) | ★ | ★ | ★ | ★ | — | — | ★ | ★★★ | 60% |
| Zarkovic et al. (2020) | ★ | ★ | ★ | ★ | ★ | ★ | — | ★★★★ | 80% |
| Buitrago et al. (2020) | ★ | ★ | ★ | ★ | — | — | ★ | ★★★ | 60% |
| Chang et al. (2021) | ★ | ★ | ★ | ★ | — | ★ | ★ | ★★★★ | 80% |
| Kim et al. (2021) | ★ | ★ | ★ | ★ | — | ★ | — | ★★★ | 60% |
| Diot et al. (2021) | ★ | ★ | ★ | ★ | — | — | ★ | ★★★ | 60% |
| Žarković et al. (2021) | ★ | ★ | ★ | ★ | — | ★ | ★ | ★★★★ | 80% |
| Conner et al. (2021) | ★ | ★ | ★ | ★ | — | ★ | ★ | ★★★★ | 80% |
| Bajpai et al. (2021) | ★ | ★ | ★ | ★ | ★ | ★ | ★ | ★★★★★ | 100% |
| Flux et al. (2023) | ★ | ★ | ★ | — | ★ | — | ★ | ★★★ | 60% |
| Lu et al. (2024) | ★ | ★ | — | ★ | ★ | — | ★ | ★★★ | 60% |
| Cumplido-Trasmonte et al. (2024) | ★ | ★ | ★ | — | ★ | ★ | ★ | ★★★★ | 80% |

**Note：S1: Is there a clearly defined research question? S2: Is the collected data sufficient to answer the research question?**

**Q1 = Is the sampling strategy relevant to address the research question?
Q2 = Is the sample representative of the target population?
Q3 = Are the measurements appropriate?
Q4 = Is the risk of nonresponse bias low?
Q5 = Is the statistical analysis appropriate to answer the research question?**
