# Supplementary material for: A scoping review of the application scope of digital technologies in lower limb rehabilitation and balance training for children with cerebral palsy
Source: Front Pediatr. 2026 Mar 18;14:1786311. doi: 10.3389/fped.2026.1786311 (PMC13039068; doi:10.3389/fped.2026.1786311)
Supplement: Supplementary file 2 [file Supplementaryfile2.docx]

# Appendix 2 – Search Strategy

Pubmed

| **1** | Cerebral Palsy[MeSH Terms] | 24902 |
| --- | --- | --- |
| **2** | ((((((CP[Title/Abstract]) OR (Spastic Diplegia[Title/Abstract])) OR (Atonic Cerebral Palsy[Title/Abstract])) OR (Hypotonic Cerebral Palsy[Title/Abstract])) OR (Dyskinetic Cerebral Palsy[Title/Abstract])) OR (Dystonic-Rigid Cerebral Palsies[Title/Abstract])) OR (Mixed Cerebral Palsies[Title/Abstract]) | 84178 |
| **3** | 1 OR 2 | 101091 |
| **4** | (Child[MeSH Terms]) OR (Disabled Children[MeSH Terms]) | 2243955 |
| **5** | ((Handicapped Children[Title/Abstract]) OR (Children with Disabilities[Title/Abstract])) OR (Children[Title/Abstract]) | 1349986 |
| **6** | 4 OR 5 | 2626559 |
| **7** | 3 AND 6 | 21971 |
| **8** | (((((((Digital Technology[MeSH Terms]) OR (Internet[MeSH Terms])) OR (Virtual Reality[MeSH Terms])) OR (Exergaming[MeSH Terms])) OR (Computers[MeSH Terms])) OR (Wearable Electronic Devices[MeSH Terms])) OR (Robotics[MeSH Terms])) OR (Artificial Intelligence[MeSH Terms]) | 447204 |
| **9** | ((((((((((((((((((Information[Title/Abstract] AND Communication Technology[Title/Abstract]) OR (ICT[Title/Abstract])) OR (VR[Title/Abstract])) OR (AI[Title/Abstract])) OR (Web[Title/Abstract])) OR (Cyberspace[Title/Abstract])) OR (Instructional Virtual Reality[Title/Abstract])) OR (Virtual Reality Exercise[Title/Abstract])) OR (Active-Video Gaming[Title/Abstract])) OR (Digital Computer[Title/Abstract])) OR (Wearable Devices[Title/Abstract])) OR (Wearable Technology[Title/Abstract])) OR (Socially Assistive Robots[Title/Abstract])) OR (Telemedicine[Title/Abstract])) OR (Telehealth[Title/Abstract])) OR (mHealth[Title/Abstract])) OR (Intelligent Robot[Title/Abstract])) OR (Platform[Title/Abstract])) OR (Videoconferencing[Title/Abstract]) | 604733 |
| **10** | 8 OR 9 | 976143 |
| **11** | 7 AND 10 | 897 |
