# Supplementary material for: A scoping review of the application scope of digital technologies in lower limb rehabilitation and balance training for children with cerebral palsy
Source: Front Pediatr. 2026 Mar 18;14:1786311. doi: 10.3389/fped.2026.1786311 (PMC13039068; doi:10.3389/fped.2026.1786311)
Supplement: Supplementary file 3 [file Supplementaryfile3.docx]

**Appendix 3. Summary of included studies.**

N/A= not available, A= Non-randomized studies, B= Randomized controlled trials, I= Intervention group, C= Control group.

| **Author(s)(year)**  **Country** | **Research designs** | **Study subjects** | **Technology type** | **Intervention measures** | | | **Frequency and duration** | **Main research results** |
| --- | --- | --- | --- | --- | --- | --- | --- | --- |
|  |  |  |  | **I** |  | **C** |  |  |
| Deutsch et al. (2008)  USA | A (Case Report) | 1 adolescent (13 years old, GMFCS Level III, spastic diplegia) | VR | Rehabilitation training using Wii VR sports games (tennis, boxing, bowling, golf). |  | N/A | The training was conducted 2-3 times per week, with each session lasting 60-90 minutes, totaling 11 training sessions | Through Wii gaming training, the patient showed improvements in visual-perceptual processing, postural control, and functional mobility, with a particularly significant enhancement in visual-perceptual resolution. |
| Meyer-Heim et al. (2009)  Switzerland,Germany | A(Single-group pre–post experimental study) | 22 children with CP (mean age 8.6 years, GMFCS Levels II-IV) | Robots and Exoskeletons | DGO robot-assisted treadmill training. |  | N/A | The training was conducted 3 to 5 times per week, with each session lasting 45 to 60 minutes, over a period of 3 to 5 weeks | The intervention led to a significant improvement in gait speed, and there was a notable increase in the standing dimension of the Gross Motor Function Measure-66 (GMFM-66). Additionally, while scores for the 6-Minute Walk Test (6MinWT), Functional Ambulation Classification (FAC), and the walking dimension of the GMFM also showed improvement, these changes did not reach statistical significance. |
| Wu et al. (2010)  USA | A(Single-group pre–post intervention study) | 12 children with CP(aged 5-15 years, GMFCS Levels I-Ⅲ) | Robots and Exoskeletons | Passive stretching and active movement training were conducted using a portable robot. |  | N/A | The training was conducted 3 times per week over a period of 6 weeks | Significant improvements were observed in both passive and active range of motion (ROM), selective motor control, and overall motor function. |
| Sandlund et al. (2011)  Sweden | A(Single-group pre–post feasibility study) | 14 children with CP (aged 6-16 years, GMFCS Levels I-III) | Games | Interactive gaming using Playstation 2 and EyeToy. |  | N/A | ≥20 min/day (mean 33 min/day) for 4 weeks | Motor performance improved, activity levels increased, and motivation was enhanced post-intervention. Both parents and children showed high levels of engagement. However, no significant progress was observed in the 1-minute walk test (1MWT) and Bruininks-Oseretsky Test of Motor Proficiency, 5th edition, subtest 5:6 (BOTMP 5:6). |
| Bilde et al. (2011)  Denmark | A(Single-group pre–post pilot study) | 9 children with CP (aged 6-13 years) | Internet | A personalized home-based training program was delivered via the internet, incorporating cognitive, perceptual, and motor skills training. |  | N/A | The training was conducted for 30 minutes daily over a period of 20 weeks | Significant improvements in motor skills (AMPS), functional strength, endurance, and visual-perceptual skills. |
| Brien et al. (2011)  Canada | A(single-subject multiple-baseline / ABA) | 4 adolescents with CP (aged 13-18 years, GMFCS Level I) | VR | Balance training was conducted using the IREX VR system. |  | N/A | Two 45-minute sessions per day for 5 consecutive days | The VR intervention significantly improved balance function and gait ability in adolescents with CP, and these effects were maintained one month later. |
| Clanchy et al. (2011)  Australia | A(**Cross-sectional validation study)** | 30 children and adolescents with CP (mean age 12.6 ± 2.0 years) | Sensors and Feedback | ActiGraph accelerometers and Cosmed K4b2 were worn to assess activity intensities (sitting, comfortable walking, brisk walking, and fast walking). |  | N/A | Each activity was performed for a specific duration (based on gait speed) | The ActiGraph was able to accurately distinguish between different gait intensities and estimate the duration of moderate-to-vigorous physical activity. |
| Jelsma et al. (2012)  South Africa | A(Multiple baseline single-case experimental design**)** | 14 children with spastic hemiplegia (aged 7-14 years) | VR | VR balance and movement training was conducted using the Nintendo Wii Fit. |  | N/A | Three sessions per week, each lasting 20 minutes, over a period of 3 weeks | Balance scores showed significant improvement, while changes in respiratory sinus arrhythmia (RSA) and timed up and down stairs test (TUDS) did not reach statistical significance. |
| Gordon et al. (2012)  Jamaica | A(**Pilot pre–post study**) | 7 children with dyskinetic CP (aged 6-12 years) | VR | Rehabilitation training was conducted using VR games on the Nintendo Wii (Wii Sports Boxing, Baseball, and Tennis). |  | N/A | The training was conducted twice a week, with each session lasting 45 minutes, over a period of 6 weeks | Significant improvement in GMFM scores following Wii training. |
| Ramstrand et al. (2012)  Sweden | A(Non-randomized controlled feasibility study) | 18 children with unilateral or bilateral CP (mean age approximately 11 years, GMFCS Levels I-II) | VR | An intervention was conducted using Wii Fit VR balance games. |  | N/A | The training was conducted five times per week, with each session lasting 30 minutes, over a period of 5 weeks | The intervention group showed minor improvements in balance during the testing period, but no statistically significant differences were observed in the test results. |
| Sharan et al. (2012)  India | B | 8 intervention, 8 control. aged 5-10 years, with CP (GMFCS Levels I-IV) | VR | VR training was conducted using Wii Fit and Wii Sports. |  | Conventional therapy | Every 3 alternate days per week for 3 weeks | The intervention group showed significant improvements in balance ability and manual dexterity. Engagement, motivation, cooperation, and child satisfaction were also significantly higher in the intervention group compared to the control group. |
| Chen et al. (2012)  China | B | 27 children with spastic CP (aged 6-12 years) | VR | A virtual cycling training system was used to strengthen lower limb muscles and enhance bone density. |  | Conventional activities | 12 weeks home-based virtual cycling training | The virtual cycling training group showed significant improvements in femoral bone density and strength of knee extension and flexion. |
| Burdea et al. (2013)  USA | A(**Case series**) | 3 children with CP (aged 7-12 years, male) | Robots and Exoskeletons | Training for ankle strength, motor control, and coordination was conducted using the RA CP robot combined with VR games. |  | N/A | Three sessions per week over a period of 12 weeks, totaling 36 training sessions | Ankle function improved to varying degrees in all three participants, with significant increases in gait speed and coordination. Game performance demonstrated enhanced ankle control and endurance. |
| Druz Bicki et al. (2013)  Poland | B | 52 children with spastic diplegic CP (ages 6-13) | Robots and Exoskeletons | Using the Lokomat robotic orthosis combined with individualized exercise training |  | Only individualized exercise training | 20 treatment sessions | No significant between-group differences in most gait parameters. |
| Radtka et al. (2013)  USA | A(Pilot feasibility study ) | 14 children with CP (ages 7-14, GMFCS levels I-III) | Games | Using video game-based physical therapy |  | N/A | N/A | Video game training enhanced the appeal of balance training, with the gaming system performing well in terms of safety and usability, making it suitable for children to use at home. |
| Luna-Oliva et al. (2013)  Spain | A(**Single-group pre–post study with follow-up**) | 11 children with CP (average age 7.91 years, GMFCS levels I-II) | VR | Using Xbox 360 Kinect for VR gaming therapy, combined with conventional physical therapy |  | N/A | Twice a week, 30 minutes per session, for a duration of 8 weeks | Significant group-level improvements were observed in GMFM, AMPS, PRT and 10MWT, maintained at 8-week follow-up. |
| Brégou Bourgeois et al. (2014)  Switzerland | A(**Case-control study**) | 14 children with CP (ages 6-15) and 15 healthy control children (ages 6-15) | Sensors and Feedback | Using foot-worn inertial sensors to analyze spatiotemporal gait parameters, assessing straight-line gait and turning gait |  | N/A | Single gait assessment session (~20 min) | Children with CP exhibited a longer stance phase and shorter swing phase, with significantly lower gait speed and step length compared to healthy controls. Gait changes were more pronounced during turning. |
| Pavão et al. (2014)  Brazil | A(**Case report**) | 1 seven-year-old boy, GMFCS level I, with spastic hemiplegia | VR | Using XBOX®360 Kinect for VR therapy |  | N/A | Twice a week, 45 minutes per session, for a total of 12 sessions | Pediatric Balance Scale (PBS) increased by 3 points; improvements were also observed in eye-hand coordination and manual dexterity. |
| Ballaz et al. (2014)  Canada | A(Observational cross-sectional comparative study) | 10 children with CP (ages 7-12, spastic diplegia, GMFCS levels I and II, 4 boys and 6 girls), and 10 typically developing children of the same age (ages 7-12, 5 boys and 5 girls) | VR | Using Wii Fit VR game |  | N/A | Approximately 10 min skiing game assessment (last slalom analyzed) | Children with CP performed poorly in visually guided weight shifting, showing smaller center of pressure (COP) range and velocity, with poorer trunk and lower limb coordination. The COP range and velocity were significantly lower than those of typically developing children (*P*<0.05). |
| Chen et al. (2014)  USA | A(**Single-group pre–post study with follow-up)** | 23 children with CP (ages 5-17, 12 boys and 11 girls) | Robots and Exoskeletons | Home-based remote assistance robot for ankle joint extension and active exercise training |  | N/A | Three times a week, 46 minutes per session, for a duration of 6 weeks | Ankle joint passive and active range of motion increased, muscle strength improved, spasticity reduced, and balance and selective control were enhanced. |
| Collange Grecco et al. (2015)  Brazil | B | 20 children with CP (ages 5-10, GMFCS levels II-III) | VR | VR gait training combined with anodal tDCS |  | VR gait training combined with sham tDCS | Five times a week, 20 minutes per session, for a duration of 2 weeks | Significant improvements in gait velocity, cadence, gross motor function (GMFM D and E), and mobility in the experimental group. Increased motor cortex excitability post-treatment but not maintained at 1-month follow-up. |
| Bingham et al. (2015)  USA | A(Cross-sectional correlational study) | 15 children with CP (ages 4-14, 9 girls and 6 boys, primarily spastic CP) | Games | Using the Balance Master postural recording game to assess balance control ability and its correlation with GMFM in children with CP |  | N/A | One 15-minute session (three balance games) | Posturography game performance, especially directional control measures, was significantly correlated with GMFM standing and overall scores. |
| van Gelder et al. (2016)  Netherlands | A(Within-subject repeated-measures study) | 16 children with CP (ages 6-16, GMFCS levels I-III) | Sensors and Feedback | Real-time visual feedback on hip or knee kinematics during treadmill walking in a virtual reality environment |  | Treadmill walking in virtual reality without real-time feedback | Three walking conditions (NF, FH, FK), each lasting 2 minutes during a single laboratory session | Most participants (except for one) were able to improve knee or hip extension under feedback, with 9 participants showing clinically relevant improvements. |
| Tarakci et al. (2016)  Turkey | B | 38 children with CP (5–18 years; GMFCS I–III; 30 completed) | VR | Combined with conventional therapy and Wii Fit video game |  | Only conventional therapy | Twice weekly, 50 minutes per session, for 12 weeks | Both groups improved; Wii group demonstrated significantly greater improvements in balance measures (FRT, STS, TUG, 10mWT, 10SCT) and total Wee FIM score compared with control. |
| Saxena et al. (2016)  India | B | 14 children with CP (average age 10.31 years, GMFCS levels Ⅱ-III) | Sensors and Feedback | Computer-based balance training with visual feedback (BTVF) |  | Usual physiotherapy only | 2 sessions/day, 15 minutes per session, for 2 consecutive days (4 sessions total) | BTVF was feasible and safe (100% retention, no serious adverse events). No significant between-group differences were observed in postural sway measures. |
| Pu et al. (2016)  China | A(Within-subject repeated-measures study) | 13 children with spastic diplegia and dynamic equinus (ages 4-11, GMFCS I–II) | Sensors and Feedback | Real-time feedback training, using DFPI (Dynamic Foot Pressure Imaging) as feedback for gait training |  | Conventional gait training | N/A | Real-time feedback training significantly improved gait quality, particularly in heel strike impact and normal gait percentage, with feedback training outperforming conventional gait training methods. |
| Mitchell et al. (2016)  Australia | B | 102 children with unilateral CP (ages 8-17, GMFCS I–II) | Internet | Web-based training |  | Usual care (waitlist control) | Six times a week, 30 minutes per session, for a duration of 20 weeks | Significant improvements were observed in functional strength and 6-minute walk test (6MWT), but no significant differences were found in activity performance. |
| Lee et al. (2016)  South Korea | A**(Case series)** | 2 children with CP (11-year-old boy, 11-year-old girl, GMFCS level II) | Sensors and Feedback | Using an augmented reality system combined with an infrared camera for real-time feedback in gait training |  | N/A | Twice a week, 30 minutes per session, for a duration of 2 weeks | Improvements were observed in gait velocity, step length, stride length, and Functional Ambulation Performance Score (FAPS) in both participants. |
| Chen et al. (2016)  USA | B | 41 children with CP (GMFCS I–III; Home n=23, Lab n=18) | Robots and Exoskeletons | Home-based and laboratory robot therapy, combined with passive stretching and active exercise training |  | Same robotic protocol performed in laboratory under supervision | 6 weeks, three times a week | Both groups showed significant improvements in biomechanical and clinical outcomes. No significant between-group differences were observed. Some follow-up improvements (e.g., DF PROM, stiffness, 6MWT, TUG) were better maintained in the laboratory-based group. |
| Lazzari et al. (2016)  Brazil | B | 20 children with CP (14 boys, 6 girls; mean age 7.5 years; GMFCS I–III) | VR | tDCS combined with VR training |  | VR training combined with sham tDCS | Five times a week, 20 minutes per session, for a duration of 2 weeks | Significant postintervention and follow-up effects favoring the experimental group were found for the Pediatric Balance Scale, Timed Up and Go Test, and center of pressure oscillation area (eyes open condition). |
| Cho et al. (2016)  South Korea | B | 18 children with spastic CP (GMFCS I–III; VRTT n=9, TT n=9) | VR | VR treadmill training |  | Conventional treadmill training | Three times a week, 30 minutes per session, for a duration of 8 weeks | The VRTT group showed significantly greater improvements in knee extensor strength, PBS, 10MWT, 2MWT, and GMFM standing domain compared to the TT group. No significant between-group difference was found in GMFM walking/running/jumping domain. |
| Wallard et al. (2017)  Belgium, France | B | 30 children with bilateral spastic CP, ages 8-10 years, GMFCS level II | Robots and Exoskeletons | Lokomat® Pediatric robotic therapy |  | Daily physical therapy | Five times a week, 40 minutes per session, for a duration of 4 weeks | The treated group showed significant improvements in full-body kinematics, particularly in head stabilization, reduced shoulder elevation and elbow flexion, and improved knee and ankle kinematics. Significant improvements were also observed in GMFM dimensions D (standing) and E (walking). |
| Chen et al. (2017)  USA | A(Within-subject experimental study; baseline–acquisition–extinction) | 7 children with CP (GMFCS I–III) and 10 typically developing children | Robots and Exoskeletons | Using the humanoid robot Darwin combined with VR game (Super Pop VRTM) for arm movement training, providing feedback |  | N/A | 2 baseline games (75 s each) 3 acquisition games with feedback 2 extinction games without feedback | Acquisition and extinction phases showed significantly higher percentage of successful reaches, shorter movement time, and higher average speed compared to baseline. No significant group effect was found. |
| Gatica-Rojas et al. (2017)  Chile | B | 32 children with CP (ages 7–14; mean age 10.7±3.2 years; spastic diplegia [SDI] and spastic hemiplegia [SHE]; GMFCS I–II/GMFCS-ER) | VR | Using the Nintendo Wii Balance Board for VR balance training |  | standard physiotherapy | Three times a week for 6 weeks (session duration not reported); assessed at baseline and every 2 weeks; two follow-ups over an additional 4 weeks | Compared with standard physiotherapy, Wii-therapy significantly reduced CoP sway area (CoPSway; *P*=0.02) and SDAP in the eyes-open condition (P=0.01); effects waned 2–4 weeks after the intervention; post-hoc analysis suggested benefits mainly in the SHE subgroup. |
| Lerner et al. (2017)  USA | A(Case report) | 1 six-year-old boy, GMFCS level II, with spastic diplegia | Robots and Exoskeletons | Using a powered exoskeleton for knee extension to assist in treating squat gait |  | N/A | Total walking time 18 minutes across four sessions | The powered exoskeleton increased peak knee extension during stance (up to 18.1°), reduced maximum knee flexion during stance, and significantly increased total knee range of motion. No significant decrease in knee extensor EMG activity was observed. |
| Wu et al. (2017)  USA | B | 23 children with CP (14 boys, 9 girls, average age 10.9±3.2 years, GMFCS levels I-IV) | Robots and Exoskeletons | Robot-assisted gait training, providing control over the pelvis and legs to facilitate weight shifting |  | Treadmill training with manual assistance for the legs | Three times a week, 30 minutes per session, for a duration of 6 weeks | Robotic training significantly improved self-selected walking speed (*P*=0.03) and 6MWD (*P*=0.048). Only the improvement in 6MWD was significantly greater than treadmill-only training (*P*=0.01). |
| Meyns et al. (2017)  Belgium | A(Non-randomized controlled trial; feasibility study) | 11 children with CP (ages 5-18, GMFCS levels I-IV) | VR | Adding VR training to conventional rehabilitation therapy |  | Conventional rehabilitation therapy | Intervention group:3 sessions/week, 30 minutes/session, until discharge  Control group:2 hours physiotherapy + 2 hours posture training per day, 5 days/week | Both groups showed improvements in seated balance (TCMS scores). As a pilot feasibility study, no inferential statistical comparisons were performed. VR training did not demonstrate clear additional benefits over conventional rehabilitation, and enjoyment levels were similar between groups. |
| Gagliardi et al. (2018)  Italy | A(Pilot study; single-group pre–post) | 16 children with bilateral spastic CP (mean age 11 ± 2.4 years; GMFCS I–III) | VR | VR gait and endurance training through the GRAIL platform |  | N/A | 5 sessions per week, 30 minutes per session, for 4 weeks (18 sessions total) | Significant pre–post improvements were observed in gross motor function (GMFM-88, *P*=0.041; dimensions D and E), walking endurance (6MWT, *P*=0.026), stride length (*P*=0.001–0.003), walking speed (*P*=0.001–0.002), and multiple kinematic parameters including ankle, knee, hip, and pelvic range of motion. No significant change was detected in the Functional Assessment Questionnaire (FAQ). |
| Choi et al. (2018)  South Korea | A(Quantitative non-randomized study) | 28 children with spastic CP (knee n=18; ankle n=10; GMFCS I–V) | Sensors and Feedback | IMU-based Modified Tardieu Scale (iMTS) with visual biofeedback for passive stretch velocity regulation |  | Conventional MTS (Motor Task Score) assessment | Test–retest and inter-rater reliability assessment; repeated fast and slow passive stretches; re-test within 3 days | The IMU-based system showed high accuracy (RMSE < 3°) and good test–retest and inter-rater reliability (ICC > 0.8), whereas the conventional MTS demonstrated poor to moderate reliability (ICC 0.2–0.6). The system also showed high agreement with EMG for clonus duration measurement (ICC = 0.96) |
| Chiu et al. (2018)  China | A(Single-group, pre–post intervention study) | 20 children with CP (ages 6-12) | VR | Using Wii Fit for VR balance and exercise training |  | N/A | Three times a week, 20 minutes per session, for a duration of 8 weeks, total 24 sessions | Significant pre–post improvements were observed in ankle dorsiflexor strength (MD 2.2 N·m, *P*<0.001), plantarflexor strength (MD 2.2 N·m, *P*<0.001), quadriceps strength (MD 7.8 N·m, *P*<0.001), preferred walking speed (MD 0.25 m/s, *P*=0.004), fast walking speed (MD 0.24 m/s, *P*<0.001), and 6-minute walk distance (MD 28 m, *P*=0.004). Independence in participation also improved slightly (*P*=0.04). |
| Bulea et al. (2018)  USA | A(Repeated-measures device study) | 7 children with CP (10.5 ± 4.6 years old, GMFCS levels I-III) | Robots and Exoskeletons | Overground walking with a pediatric knee extension exoskeleton providing stance and late-swing assistance |  | Baseline walking without exoskeleton | Six visits over 8–12 weeks; total overground practice time 30–65 minutes across visits | EMG variance ratio was significantly higher during exoskeleton-assisted walking compared to baseline (*P* < 0.001). VR decreased from the first to the final visit, suggesting adaptation to the device, though variability remained higher than baseline. |
| Wallard et al. (2018)  Belgium, France | B | 30 Children with CP (ages 8-10, GMFCS level II) | Robots and Exoskeletons | Using Lokomat® Pediatric for gait training |  | Daily physical therapy | 4 weeks, five times a week, 40 min/session | Significant intragroup improvements were observed in the treated group for gait speed (p=0.046), step length, cadence reduction (p=0.029), step width reduction, increased single support time, decreased double support time, and GMFM dimensions D and E. Kinetic analysis revealed significant improvements in COM–COP correlation and reduced time lag between COM–COP divergence and propulsive force generation, indicating enhanced dynamic equilibrium control. No comparable improvements were observed in the control group. |
| Hsieh et al. (2018)  China | B | 40 children with CP (ages 5-10, GMFCS II–IV) | Games | Using a PC gaming platform for balance training, combined with dynamic standing training |  | Using a computer mouse for the same game, but without a gaming platform | Five times a week, 40 minutes per session, for a duration of 12 weeks | Significant group × time interactions were found for AP center-of-pressure (CoP) sway, sway velocity, Berg Balance Scale (BBS), and Timed Up and Go (TUG) scores (*P* < 0.05). The intervention group demonstrated significant improvements in balance performance, whereas the control group showed no significant pre–post changes. No significant improvements were observed in the Fullerton Advanced Balance (FAB) scale. |
| Levac et al.(2018)  Canada | A**(Pilot non-randomized controlled trial)** | 11 children with CP (ages 7-18, GMFCS levels I-II) | VR | 1 week of VR intervention followed by 6 weeks of home-based active video game training, with remote supervision by a physical therapist |  | Only 6 weeks of home-based AVG (Active Video Game) training | 30 min/day, 5 days/week | There were no significant between-group differences. The AVG-only group demonstrated a statistically and clinically significant improvement in GMFM-CM scores following the 6-week intervention (median difference 4.5 points, *P*=0.042). The VR+AVG group showed a statistically and clinically significant decrease in 6MWT distance (median decrease 68.2 m, *P*=0.043), which returned to baseline at 2 months post-intervention. |
| Bayón et al. (2018)  Spain | A(Case series) | 4 children with CP (ages 11-18, GMFCS levels II and III, 2 boys and 2 girls) | Robots and Exoskeletons | Using the CPWalker platform for gait training, assessing gait speed, step length, muscle strength, and other indicators |  | N/A | 16 non-consecutive sessions over 8 weeks (2 sessions/week). Each session included 10–15 min warm-up, 60 min overground robotic training, and 3 min cool-down | Most participants demonstrated improvements in gross motor function (GMFM-88 dimensions D and E), 6-minute walk distance, walking speed (10MWT), and maximal isometric muscle strength. Mean velocity increased by 21.46% ± 33.79%, step length by 17.95% ± 20.45%, and strength improved substantially (up to 129% in some participants). Physiological Cost Index decreased in all participants. Some kinematic improvements (GDI, GPS) were observed but were not clinically significant in most cases. |
| Booth et al. (2019)  Netherlands | A**(Repeated-measures study)** | 22 children with CP (10.5±3.1 years old, GMFCS levels I-II, with spastic hemiplegia) | VR | Gait training through a virtual avatar in a VR environment with real-time biofeedback, focusing on step length, knee extension, and ankle force. |  | N/A | Single-session experimental protocol with randomized 2-minute biofeedback trials | Through biofeedback, children achieved significant improvements in gait: step length increased by 12.7%, knee extension improved by 7.4°, and ankle force increased by 37.7%. |
| Behboodi et al. (2019)  USA | A(Cross-sectional validation study) | 7 typically developing children (12 ± 1 years) and 5 children with spastic diplegic CP (14 ± 1 years; GMFCS II–III) | Sensors and Feedback | Two shank-mounted gyroscopes (128 Hz) connected to a rule-based real-time LabVIEW algorithm to detect all seven gait phases |  | Motion capture (gold standard) and force-sensing resistors (FSR) | Participants walked on an instrumented treadmill at self-selected speed; performance evaluated over the last 10 complete gait cycles | The system detected over 99% of gait phases relative to motion capture, and detection reliability increased to 100% in children with CP using automatic thresholding. Absolute gait phase onset detection errors were generally <100 ms, with onset RMSE ranging from 35 ms to 141 ms. Gait cycle duration RMSE averaged approximately 22 ms. The system demonstrated feasibility for real-time control of functional electrical stimulation (FES). |
| Ma et al. (2019)  China, Netherlands, New Zealand | A(Cross-sectional case-control study) | 10 children with CP (ages 6-12, 8.5±2.3 years old, GMFCS levels I-II), and 10 typically developing children | VR | Participants walked at self-selected speed under two conditions within a CAREN immersive virtual reality system: • Level treadmill walking • 10° inclined treadmill walking |  | N/A | N/A | During uphill walking, both groups decreased walking speed and stride length and increased peak pelvis tilt, hip flexion, knee flexion, and ankle dorsiflexion. CP children walked significantly slower and with shorter stride length than TD children. However, no significant between-group differences were observed in medial–lateral COM–COP separation, indicating similar dynamic balance control. Uphill walking magnified pre-existing abnormal gait patterns in the CP group. |
| Yazıcı et al. (2019)  Turkey | A(Prospective non-randomized controlled trial) | 24 children with unilateral spastic CP (ages 5-12, GMFCS levels I-II) | Robots and Exoskeletons | Robotic gait training using the Innowalk Pro device combined with standard physiotherapy rehabilitation |  | Standard physiotherapy rehabilitation only | 30 minutes per session, 3 sessions per week, for 12 weeks | The intervention group showed significant improvements in balance, gait speed, functional muscle strength, and oxygenation levels, with the effects sustained for up to 3 months. |
| Mills et al. (2019)  Canada | A(Non-randomized controlled trial) | 11 children and adolescents with CP (7–17 years; GMFCS I–II) | VR | 1-hour one-on-one physiotherapist-supervised VR balance training (IREX system) |  | No intervention between assessments | 1 hour per day, for 5 consecutive days | No consistent or statistically significant differences in change scores were observed between intervention and control groups in postural control mechanisms, EMG activity, stepping responses, 6MWT, or GMFM-CM. The 5-day VR intervention did not significantly alter anticipatory or reactive postural control mechanisms. |
| Arnoni et al. (2019)  Brazil | B | 15 children with mild spastic hemiplegic CP (GMFCS I–II; mean age 10.0 ± 3.0 years) Randomized, single-blind controlled trial | VR | Conventional neurodevelopmental therapy (twice/week, 50 min)  Virtual reality training using Kinect Adventures (Xbox 360 Kinect) 45 minutes per session, twice/week, for 8 weeks |  | Conventional neurodevelopmental therapy only | Twice a week, 45 minutes per session, for a duration of 8 weeks | The intervention group showed significant improvements in GMFM Dimension D (*P*=0.021) and Dimension E (*P*=0.008), with clinically meaningful percentage changes (D=10.8%, E=14.0%). No significant changes were observed in postural sway variables for either group. |
| Mataki et al. (2020)  Japan | A(Single-session pre–post study) | 19 patients with spastic cerebral palsy (mean age 15.7 years; GMFCS I–IV) Single-group pre–post study | Robots and Exoskeletons | Single 20-minute session of Hybrid Assistive Limb (HAL) robot-assisted gait training |  | N/A | One session, 20 minutes | Walking speed significantly increased (*P*=0.003), along with right (*P*=0.020) and left (*P*=0.014) stride length. Significant improvements were observed in specific joint angles, including increased hip flexion at mid-swing, knee flexion at terminal stance, plantarflexion at mid-stance, and trunk anterior tilt during terminal swing. Joint-angle symmetry improved across multiple gait phases. The double-support period significantly decreased, while the swing phase duration increased. Gait analysis was performed only in GMFCS I–III participants (n=14). |
| Kuroda et al. (2020)  Japan | A(Case report) | 1 child with CP (11 years old, GMFCS level IV) | Robots and Exoskeletons | Using the 2S-HAL robot for gait training |  | N/A | 12 sessions over 4 weeks (2–4 sessions per week), 20 minutes per session | After the 2S-HAL intervention, walking speed, step length, cadence, and 6MWD increased, while PCI decreased. GMFM and COPM scores also improved. Several improvements were maintained at 3-month follow-up, although fluctuations were observed across time points. |
| Hsieh et al. (2020)  China | B | 56 children with CP (ages 6-10, GMFCS levels I-III) | Games | Gaming balance board training using ankle plantarflexion/dorsiflexion to control PC games |  | PC games played using a computer mouse in standing position | Three times a week, 45 minutes per session, for a duration of 12 weeks | ANCOVA revealed significant between-group improvements favoring the balance board group in CoP sway path (*P*=0.011) and sway area (*P*=0.001). Significant improvements were also observed in PBS (static, dynamic, total scores) and 2MWT (*P*=0.002). No significant between-group difference was found for CoP sway velocity. |
| Kawasaki et al. (2020)  Japan | B | 10 children with CP (ages 5-16, GMFCS levels I-III) | Robots and Exoskeletons | Using Honda Walking Assist (HWA) for gait training |  | Performing gait training without the use of a robot | Participants completed two separate experimental sessions (crossover design). Each session included: • Pre overground walking assessment • 10 × 30-second treadmill walking trials (with or without assistive torque) • Washout walking trials • Post overground assessment | Significant ASSIST × TIME interactions were observed for maximum hip flexion (p=0.009) and hip extension (*P*=0.005). Limb symmetry significantly improved after RAGT (*P*<0.05) but not after NAGT. The propulsion force (Fy2) of the affected limb increased after RAGT. No significant changes were observed in overground gait speed. |
| Jin et al. (2020)  South Korea | B | 20 children with cerebral palsy (mean age 6.75 ± 2.15 years; GMFCS II–IV) | Robots and Exoskeletons | Robot-assisted gait training (RAGT) using Walkbot-K |  | Standard care | 3 sessions per week, 30 minutes per session, for 6 weeks (18 sessions total), combined with continued standard care | Significant treatment effects were observed during the RAGT period compared with the standard care period, with improvements demonstrated in GMFM-88 Dimension D (*P*=0.018) and Dimension E (*P*=0.021), WeeFIM mobility subtotal scores (*P*=0.007), as well as COPM performance (*P*<0.001) and satisfaction scores (*P*=0.001). |
| Zarkovic et al. (2020)  Slovenia, Czechia | A(Single-arm pre–post pilot study) | 12 ambulatory children with spastic diparesis (mean age 10.9 ± 3.3 years; GMFCS I–III) | Robots and Exoskeletons | Robot-assisted gait training |  | N/A | 4 weeks, five times a week | Significant improvements were observed in selective voluntary motor control (SCALE), 6-minute walk distance (mean increase ≈75 m), and GMFM-D and GMFM-E scores, along with reduced hip flexion contracture and internal hip rotation, while 10-meter walk test speed did not significantly change; these gains were maintained at 3-month follow-up without significant decline. |
| Sucuoglu et al. (2020)  Turkey | A(a retrospective study) | 38 children with cerebral palsy (aged 4–18 years; GMFCS I–V) completed the study | Robots and Exoskeletons | Combined conventional therapy with robot-assisted gait training |  | Conventional treatment | Three to four times a week, 60 minutes per session, for a duration of 8-10 weeks | Both mild-to-moderate (GMFCS I–III) and severe (GMFCS IV–V) groups showed significant improvements in GMFM-66 Dimensions D and E (*P*<0.01), while significant improvements in 10-meter walk test and 6-minute walk test were observed only in the mild-to-moderate group, and between-group comparisons demonstrated significantly greater gains in standing and walking abilities in the mild-to-moderate group compared with the severe group (*P*<0.01). |
| Buitrago et al. (2020)  Colombia | A(Case report) | 1 eight-year-old boy with spastic CP (GMFCS level III) | Robots and Exoskeletons | Gait training and goal-directed therapy were conducted using the NAO robot, combined with SMART goal setting. |  | N/A | Two sessions per week, each lasting 45 minutes, over a period of 8 weeks | With motivation from the socially assistive robot NAO, the children showed improvements in gait and achieved the predetermined step count goals. |
| Barreira et al. (2020)  Brazil | A(Validation study) | 11 children with CP (aged 9-18 years, GMFCS Levels II-III) | Sensors and Feedback | Gait parameter assessment was conducted using Kinect v2 |  | Video recording for comparison. | Each participant underwent gait assessment three times | The Kinect v2 system effectively assessed gait parameters in children with CP, and the results were consistent with video recordings, demonstrating high accuracy. |
| Jung et al. (2020)  South Korea | B | 10 adolescents with spastic diplegic CP (aged 11-17 years, GMFCS Levels I-II) | VR | Motor training was conducted using Xbox Kinect, combined with soccer and beach volleyball games. |  | Conventional Treatment | Three sessions per week, each lasting 40 minutes, over a period of 6 weeks | The Kinect video game training group showed significant improvements in SCALE assessments (except for right hip abduction) and Pediatric Balance Scale (PBS) scores. |
| Chang et al. (2021)  South Korea | A(Single-group pre–post pilot study) | 16 children with CP (aged 5 - 17 years, GMFCS level I - IV) | VR | Use VR combined with an equestrian simulator for training |  | N/A | 2 sessions per week, 30 minutes per session, lasting for 8 weeks | The intervention group showed significant improvements (*P*<0.05) in Pediatric Balance Scale (PBS), total scores of Gross Motor Function Measure-66 (GMFM-66) and Gross Motor Function Measure-88 (GMFM-88), as well as in Dimension D (standing) and Dimension E (walking, running, and jumping) of GMFM-88. |
| Kim et al. (2021)  South Korea | A(validity study) | 12 children with cerebral palsy (7 boys, 5 girls; mean age 9.0 ± 2.1 years; age range 6–15 years; GMFCS I–III) | Sensors and Feedback | Use a smartphone application to assess pelvic rotation during sitting, standing, and single - leg standing. |  | Use a palpation meter to conduct the same assessment of pelvic rotation | Retest will be carried out two weeks later | The smartphone measurement of pelvic rotation has good test - retest reliability. Moreover, there is a significant linear correlation between the measurement results of the smartphone and those of the palpation meter. |
| Bajpai et al. (2021)  India | A(Algorithm development and validation study) | 356 children with cerebral palsy (age 4–18 years; GMFCS I–III) and 41 typically developing children from publicly available gait databases. | Sensors and Feedback | Use A - GAS to evaluate gait abnormalities, including calculating knee joint angles, hip joint angles, etc. |  | N/A | Automated analysis of each gait cycle | The A-GAS values clearly differentiated CP from typically developing subjects with minimal overlap in probability distributions, and a decision threshold around 0.13 allowed accurate classification; Bland–Altman analysis showed that the three-joint configuration demonstrated 96% agreement within the 95% confidence interval compared to the nine-joint configuration, indicating comparable performance between low-cost and high-cost assessment setups |
| Kim et al. (2021)  South Korea | A(Case series) | 3 children with CP (aged 9 - 16 years, GMFCS II - IV) | Robots and Exoskeletons | Wear the Angel - legs robot for gait training |  | N/A | Conduct the training 1 to 3 times a week, 60 minutes each time, lasting for 7 to 20 weeks | All participants showed significant improvements in Gross Motor Function Measure (GMFM), gait speed, gait endurance, and oxygen consumption efficiency. In particular, children at GMFCS levels II and III demonstrated remarkable improvements in gait speed and gait endurance. |
| Diot et al. (2021)  Canada | A(Case report) | One 7 - year - old female patient with CP (GMFCS level V, accompanied by lower limb muscle spasm and dyskinesia) | Robots and Exoskeletons | Use the Trexo Home robotic gait trainer for gait training. |  | N/A | On average, it was used 3.3 days per week, mean usage time 46 min/week, for a total of 12 weeks | After using the Trexo Home robotic gait trainer, the participants experienced an increase in defecation frequency, and improvements in head control and knee flexor spasm. However, no consistent improvements were observed in postural stability, ROM, and other muscle spasms. |
| Žarković et al. (2021)  Czechia, Slovenia | A(Single-group pre–post study) | 12 Children with CP (aged 10.4 years, GMFCS Levels I-III) | Robots and Exoskeletons | Conduct 20 gait training sessions using the Lokomat Pro. |  | N/A | Over a period of 4 weeks, with 5 sessions per week | After the 4-week RAGT program, significant pre–post improvements (*P*< 0.01) were observed in decreased muscle activity of biceps femoris, rectus femoris, and tibialis anterior, reduced internal hip rotation range, increased cadence and step length, and decreased double support time and stride time, while no significant changes were found in kinetic gait variables. |
| Conner et al. (2021)  USA | A(Single-group pre–post pilot study) | 5 children with CP (aged 12 - 17 years, GMFCS I - II, two with bilateral CP and three with unilateral CP) | Robots and Exoskeletons | wearable ankle exoskeleton providing resistance proportional to biological ankle moment |  | N/A | Ten 20-minute treadmill walking sessions separated by 48–72 hours | After training, participants showed significant improvements in gait control: reduced muscle co-contraction, increased neural control complexity, and optimized gait efficiency and metabolic consumption. |
| Farr et al. (2021)  Britain | B | 30 children with ambulatory CP (GMFCS I–II), aged 5–16 years (21 completed the study) | VR | A personalized VR activity program supported by a physical therapist |  | Freely chosen Wii Fit games | Three times a week, 30 minutes per session, for 12 weeks | Both groups showed improvements in GMFM-66 and TUG scores, with small between-group differences; GAS scores improved notably; VR therapy was feasible and acceptable, but definitive clinical superiority was not established. |
| Gercek et al. (2022)  Turkey | B | 19 children with unilateral CP (ages 6-12, GMFCS I-II) | VR | Virtual golf training |  | Traditional golf training, three times a week, 60 minutes per session, for 12 weeks | Three times a week, 60 minutes per session, for 12 weeks | Both training methods significantly improved gait, muscle strength, flexibility, and aerobic endurance. Virtual golf training outperformed traditional training in balance control, while traditional golf training showed significant effects in reducing muscle spasticity. |
| De Luca et al. (2022)  Italy | A(Non-randomized pre-post study) | 10 children with CP (ages 6-12, ASCP,) | Robots and Exoskeletons | Gait training using the Lokomat robot, combined with conventional physical therapy |  | N/A | Twice a week, 45 minutes per session, for 3 months | Significant improvements were observed in GMFM total score (*P*<0.001), particularly in sitting and walking domains, and in CP-QOL (*P*<0.005). No control group was included. |
| Jung et al. (2022)  South Korea | A(Non-randomized controlled trial) | 17 children with CP (ages 5-18, GMFCS I-IV) | VR | VR horseback riding simulator training |  | Home-based aerobic exercise | Twice a week, 30 minutes per session, for 16 sessions | The intervention group showed significant improvements in Gross Motor Function Measure (GMFM) scores and body composition (Fat-Free Mass [FFM], Skeletal Muscle Mass [SMM]), while the control group showed no significant changes. |
| Moll et al. (2022)  Germany | B | 30 Children with CP, 25 completed analysis. (average age 13 years, GMFCS II + III) | Robots and Exoskeletons | Gait training using the Hybrid Assistive Limb (HAL) system, supplemented with physical therapy, massage therapy, and other interventions |  | Receiving only conventional physical therapy and other interventions | 11-day inpatient treatment, with multiple interventions provided daily | The intervention group showed significant improvements in Gross Motor Function Measure (GMFM) total score and GMFM Dimensions D (Standing) and E (Walking, Running, and Jumping), but no significant changes were observed in the 10-Meter Walk Test (10MWT) and 6-Minute Walk Test (6MWT). |
| Flux et al. (2023)  Netherlands | A(Observational cross-sectional feasibility study) | 18 children with CP (ages 6-17, GMFCS I-II) | VR | Gait training on a treadmill, combined with electromyographic (EMG) feedback-driven VR games to modify the activation patterns of the calf muscles |  | N/A | Single intervention session, lasting 18 minutes | Participants significantly decreased early stance EMG activity (−6.8%, p=0.025) and increased push-off activity (+8.1%, p=0.039), but no significant change in double-bump-index. Peak ankle power increased modestly. No functional outcomes were assessed. The intervention was highly enjoyable. |
| Grodon et al. (2023)  Britain | A(Single-group pre–post study) | 27 children with CP (ages 5-18, GMFCS IV/V) | Robots and Exoskeletons | Gait training using a robotic rehabilitation device (Innowalk Pro, IP) |  | N/A | Four times a week, 30 minutes per session, for 6 weeks | The intervention group showed significant improvements in quality of life (QoL) and functional goals (GAS), especially among participants in the middle school age group. In terms of range of motion (ROM), knee extension improved significantly, but these improvements were not maintained after 3 months. |
| Lee et al. (2023)  USA | A(Single-group pre–post pilot study) | 8 children with CP (8.1 ± 2.6 years old, GMFCS I-III) | Robots and Exoskeletons | Rehabilitation training using an ankle rehabilitation robot |  | N/A | Three times a week, 40-50 minutes per session, for 6 weeks | Significant improvements were observed in dorsiflexion strength (p=0.018), plantarflexion strength (*P*=0.043), dorsiflexion AROM (*P*=0.028), PBS (*P*=0.026), TUG (*P*=0.018), and 6MWT (*P*=0.018). Proprioceptive acuity showed a non-significant trend toward improvement. |
| Choi et al. (2024)  South Korea | B | 90 children with CP (ages 6-15, GMFCS II-IV) | Robots and Exoskeletons | Robot-assisted gait training (RAGT) |  | Conventional physical therapy | Three times a week, 30 minutes per session, for 6 weeks | RAGT produced significantly greater improvements in GMFM-88 total, GMFM-E, and GMFM-66 at post-intervention. At 4-week follow-up, improvements were maintained for GMFM-88, balance control (PBS), and gait deviation index (GDI). No significant between-group differences were observed in 6MWT distance or gait speed. |
| Avaltroni et al. (2024)  Italy | A(Observational pre–post study) | 22 children with CP (ages 2-8, Predominantly GMFCS IV–V [with a few III and II]) | Robots and Exoskeletons | Gait and standing training using the Moonwalker robotic exoskeleton |  | N/A | 20 sessions in clinic, continued home use for ~5 months | Exoskeleton-assisted walking speed (10MWT) significantly increased from T0 to T1 and further improved at T2. At follow-up, most children achieved speeds of 0.4–0.8 m/s while using the device. Limited improvements in trunk sitting control (LSS) were observed in a subset (n=7). Parents reported high satisfaction (QUEST mean device score 3.9; service score 4.8). |
| Lu et al. (2024)  China, Serbia | A(Observational pilot study) | 2 children with CP (age unspecified) | VR | Rehabilitation training using a virtual robot, with training content including body movements, social interaction, and non-verbal communication |  | N/A | The data was not clearly described, with the test type focusing on social interaction and motor rehabilitation training | A virtual robot platform was used to facilitate motor tasks, social interaction, and non-verbal communication training. No standardized clinical outcome measures were reported. Observational findings suggested increased engagement and repetitive motor participation. The study primarily demonstrated technical feasibility rather than clinical effectiveness. |
| Behboodi et al. (2024)  USA | A(Case study) | 1 child with CP (11.5 years old, GMFCS level I) | Sensors and Feedback | BCI-NFT training, combining EEG detection with NMES, to train ankle dorsiflexion |  | N/A | Once a week, for a total of 10 sessions, with 100 repetitions per session | Post-training, seated dorsiflexion velocity significantly increased (*P*=0.001). However, improvements did not transfer to ankle kinematics during walking. Gait speed and step length increased descriptively, with significant improvement in right step length at self-selected speed (p=0.001). EEG analysis demonstrated increased beta-band ERD in the central motor region. Detection accuracy averaged 79%, indicating good system feasibility. |
| Hui et al. (2024)  China | B | 40 children with CP (ages 3-10, GMFCS I-III) | Robots and Exoskeletons | 40 children with CP (ages 3-10, GMFCS I-III) |  | Conventional rehabilitation training | 5 sessions/week for 8 weeks (40 sessions total); SRE group replaced one 30-min physical therapy session per day with 30-min SRE-assisted overground walking | The intervention group showed significant improvements in 10-Meter Walk Test (10MWT), 6-Minute Walk Test (6MWT), Gross Motor Function Measure Dimension D (GMFM-D), Gross Motor Function Measure Dimension E (GMFM-E), Pediatric Balance Scale (PBS), Modified Ashworth Scale (MAS), and Physiological Cost Index (PCI), with particularly notable increases in gait speed and gait distance. |
| De Mulder et al. (2024)  Belgium | A(Randomized cross-over laboratory study) | 29 children with CP (ages 9-13, GMFCS I-II) | VR | VR and verbal feedback were provided during gait training, focusing on hip extension. |  | N/A | 3-minute feedback sessions, with VR and verbal feedback provided separately during gait training | Both VB and VR feedback significantly improved minimal hip extension compared with baseline (*P*<0.001). However, no statistically significant differences were found between VB and VR feedback. Although hip extension improved, the overall Gait Profile Score did not improve and slightly worsened after VB feedback, likely due to compensatory strategies such as increased anterior pelvic tilt.. |
| Castro et al. (2024)  Spain | A(Non-randomized controlled trial) | 30 children with CP (ages 3-14, GMFCS II-Ⅴ) | Robots and Exoskeletons | Gait training using the ATLAS 2030 exoskeleton, combined with conventional rehabilitation therapy |  | Conventional rehabilitation therapy | Twice a week, 60 minutes per session, for 3 months | Compared with conventional therapy alone, the intervention group showed significantly greater improvements in GMFM-88 total score (*P*=0.012) and dimensions A and B. Significant improvements were also observed in hip, knee, and ankle ROM and in most lower limb spasticity measures. |
| Cumplido-Trasmonte et al.  (2024)  Spain | A(Prospective case series) | 6 children (3 with CP, 3 with spinal muscular atrophy, ages 3-14) | Robots and Exoskeletons | Gait training using the ATLAS 2030 exoskeleton |  | N/A | Twice per week, 60 min per session, total of 8 sessions | No serious adverse events. Progressive increases in cadence, steps, and 6MWT distance. High satisfaction reported. |
| Zhang et al. (2024)  China | B | 23 Children with CP (ages 3-10, GMFCS II-IV) | Robots and Exoskeletons | AiWalker-K robotic-assisted gait training combined with conventional rehabilitation therapy |  | Conventional rehabilitation therapy | 5 times/week, 30 minutes/session, for 4 weeks | Combination treatment showed significantly greater improvements in 6MWT, GMFM-88 (D and E), PBS, and EVGS compared with routine rehabilitation alone. No significant difference was observed in PCI. |
